# Clonal Transitions and Phenotypic Evolution in Barrett’s Esophagus

**DOI:** 10.1053/j.gastro.2021.12.271

**Published:** 2022-04

**Authors:** James A. Evans, Emanuela Carlotti, Meng-Lay Lin, Richard J. Hackett, Magnus J. Haughey, Adam M. Passman, Lorna Dunn, George Elia, Ross J. Porter, Mairi H. McLean, Frances Hughes, Joanne ChinAleong, Philip Woodland, Sean L. Preston, S. Michael Griffin, Laurence Lovat, Manuel Rodriguez-Justo, Weini Huang, Nicholas A. Wright, Marnix Jansen, Stuart A.C. McDonald

**Affiliations:** 1 Clonal Dynamics in Epithelia Laboratory, Queen Mary University of London, London, United Kingdom; 2 School of Mathematical Sciences, Queen Mary University of London, London, United Kingdom; 3 Northern Institute for Cancer Research, Newcastle University, Newcastle, United Kingdom; 4 Department of Gastroenterology, University of Aberdeen, Aberdeen, United Kingdom; 5 Department of Surgery, Barts Health NHS Trust, Royal London Hospital, London, United Kingdom; 6 Department of Histopathology, Barts Health NHS Trust, Royal London Hospital, London, United Kingdom; 7 Endoscopy Unit, Barts Health NHS Trust, Royal London Hospital, London, United Kingdom; 8 Royal College of Surgeons of Edinburgh, Edinburgh, United Kingdom; 9 Oeosophagogastric Disorders Centre, Department of Gastroenterology, University College London Hospitals, London, United Kingdom; 10 Research Department of Tissue and Energy, University College London Division of Surgical and Interventional Science, University College London, London, United Kingdom; 11 Department of Cellular Pathology, University College London Hospitals, London, United Kingdom; 12 Epithelial Stem Cell Laboratory, Centre for Cancer Genomics and Computational Biology, Barts Cancer Institute, Queen Mary University of London, London, United Kingdom; 13 UCL Cancer Institute, University College London, London, United Kingdom

**Keywords:** Barrett’s Esophagus (BE), Clonal, Esophageal Adenocarcinoma (EA), Diversity, Evolution, AP, alkaline phosphatase, BE, Barrett’s esophagus, DAB, diaminobenzidine, EA, esophageal adenocarcinoma, EGA, European Genome-Phenome Archive, EMR, endoscopic mucosal resection, FFPE, formalin-fixed, paraffin-embedded, HRP, horseradish peroxidase, IHC, immunohistochemistry, IM, intestinal metaplasia, mtDNA, mitochondrial DNA, NGS, next-generation sequencing, PCR, polymerase chain reaction

## Abstract

**Background & Aims:**

Barrett’s esophagus (BE) is a risk factor for esophageal adenocarcinoma but our understanding of how it evolves is poorly understood. We investigated BE gland phenotype distribution, the clonal nature of phenotypic change, and how phenotypic diversity plays a role in progression.

**Methods:**

Using immunohistochemistry and histology, we analyzed the distribution and the diversity of gland phenotype between and within biopsy specimens from patients with nondysplastic BE and those who had progressed to dysplasia or had developed postesophagectomy BE. Clonal relationships were determined by the presence of shared mutations between distinct gland types using laser capture microdissection sequencing of the mitochondrial genome.

**Results:**

We identified 5 different gland phenotypes in a cohort of 51 nondysplastic patients where biopsy specimens were taken at the same anatomic site (1.0–2.0 cm superior to the gastroesophageal junction. Here, we observed the same number of glands with 1 and 2 phenotypes, but 3 phenotypes were rare. We showed a common ancestor between parietal cell-containing, mature gastric (oxyntocardiac) and goblet cell-containing, intestinal (specialized) gland phenotypes. Similarly, we have shown a clonal relationship between cardiac-type glands and specialized and mature intestinal glands. Using the Shannon diversity index as a marker of gland diversity, we observed significantly increased phenotypic diversity in patients with BE adjacent to dysplasia and predysplasia compared to nondysplastic BE and postesophagectomy BE, suggesting that diversity develops over time.

**Conclusions:**

We showed that the range of BE phenotypes represents an evolutionary process and that changes in gland diversity may play a role in progression. Furthermore, we showed a common ancestry between gastric and intestinal-type glands in BE.

## Graphical abstract

**Figure:**
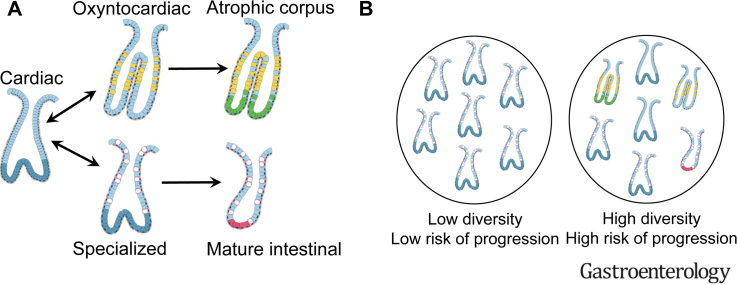

## Teaser

We do not know how Barrett’s esophagus glands evolve. A link is shown between the different types, and their diversity is associated with an increased risk of developing cancer.

See Covering the Cover synopsis on page 999.

**What You Need to Know**

**Background and Context**

We do not fundamentally understand the phenotypic evolution of Barrett’s esophagus, nor do we fully understand the distribution of gland phenotype diversity and if this is associated with progression.

**New Findings**

There is heterogeneity of Barrett’s esophagus gland phenotype close to the gastroesophageal junction that results from the evolution between gastric and intestinal gland types. Diversity of gland phenotypes is associated with progression.

**Limitations**

More longitudinal studies are needed to determine specific evolutionary steps to dysplasia.

**Impact**

This study shows for the first time how local diversity affects the Barrett’s esophagus lesion and that gastric and intestinal gland types show a common ancestry. Understanding how diversity affects patients who progress to dysplasia may prove an important predictive biomarker of cancer risk.

Barrett’s esophagus (BE) is the only known precursor condition of esophageal adenocarcinoma (EA) and is characterized by the metaplastic replacement of the normal squamous epithelium of the distal esophagus with a columnar epithelial phenotype that frequently contains intestinal metaplasia (IM).^1^ In some countries, the diagnosis of BE is based on the histopathologic presence of IM in esophageal biopsy specimens,^2^ but in others, only the endoscopic presence of columnar epithelium in the distal esophagus is required.^3^ It is widely assumed that the presence of IM assists in stratifying patients’ cancer risks, but there is evidence to suggest that this is not always the case.^4^, ^5^, ^6^ We have previously shown that glands that do not contain goblet cells can clonally expand, accumulate oncogenic *TP53* mutations, and be the source of EA.^7^ This finding highlights an important lack of understanding of the evolution of the BE epithelial phenotype.^8^^,^^9^

BE displays a rich diversity of morphologically distinct glands^10^, ^11^, ^12^, ^13^ that contain an admixture of both gastric and intestinal epithelial cell lineages.^14^ At present, we do not fully understand the scope of epithelial lineage diversity nor whether these lineages represent an evolutionary pathway that may be altered in patients who progress to dysplasia. Previous studies have documented that genotypic diversity predicts the risk of BE progressing to cancer,^15^, ^16^, ^17^ but it is currently unknown whether this is reflected in phenotypic diversity across the segment. There is controversy as to the mechanism by which genetic diversity evolves, with some data showing it is acquired over time,^18^ and others showing that diversity is inherent to BE^17^ and is always increased relative to nonprogressors.^15^ To date, the significance of the evolution of gland phenotype in BE has not been fully appreciated. This is an important omission when we consider that all current diagnoses are entirely based on histopathologic analysis^3^ and that natural selection acts fundamentally on phenotype, not genotype.^19^

Here, we address this unresolved issue by showing the frequency distribution of gland phenotypes in a cross-sectional BE patient cohort, both at a fixed point within the metaplastic segment (1.0–2.0 cm proximal of the gastroesophageal junction) and throughout the BE segment. Using mitochondrial DNA mutations as clonal marks,^20^^,^^21^ we then demonstrate ancestral clonal relationships between different gland phenotypes within nondysplastic BE, indicating phenotypic evolution. Finally, we measure gland phenotype diversity in nondysplastic BE from patients who have no history of dysplasia and compare this to BE biopsy samples taken before a diagnosis of dysplasia, nondysplastic BE adjacent to dysplasia, and postesophagectomy nondysplastic BE. Together, these data show, for the first time to our knowledge, the phenotypic evolutionary pathway within BE.

## Methods

### Patients

Patients were recruited from the surveillance BE endoscopic clinic at Barts Health National Health Service Trust and from the archives of both the Royal London Hospital and University College London Hospital approved under multicenter ethical approval from London research ethics committee (11/LO/1613 and 15/LO/2127). Postesophagectomy BE esophagus specimens were accessed under County Durham and Tees Valley 2 research ethics committee approval (08/H0908/25) and from University College London hospital (as described earlier). Snap-frozen biopsy specimens and formalin-fixed paraffin-embedded (FFPE) archival specimens were used in this study.

#### Cohort 1a

A series of 64 biopsy specimens from 51 BE patients were collected from 1.0–2.0 cm proximal of the gastroesophageal junction and were FFPE preserved. All biopsy specimens met the following inclusion criteria: (1) they were taken at the same anatomic height within the esophagus, regardless of BE maximum length; (2) they were taken from the BE lesion identified during endoscopy; and (3) no dysplasia or cancer was observed at the time of endoscopy or any history of dysplasia. The mean age of the patients within cohort 1 was 62.2 years (range, 27–89 years), the female-to-male ratio was 1:4.9, and the mean maximum BE segment length was 4.5 cm (range, 1.5–14 cm; median, 4.0 cm). For 25 of these patients, we obtained further archival FFPE H&E sections from all biopsy specimens taken at the same surveillance endoscopy. Further anonymized clinical information is detailed in Supplementary Table 1.

#### Cohort 1b

The cohort included fresh-frozen adjacent biopsy specimens taken from BE, the anatomic gastric cardia, and squamous esophagus of 20 patients. These were used for either lineage tracing or mitochondria next-generation sequencing (NGS). Further anonymized clinical information is detailed in Supplementary Table 2.

#### Cohort 2

The cohort included 99 FFPE-preserved BE biopsy specimens (19 patients) showing no dysplasia or history of dysplasia, 21 endoscopic mucosal resection (EMR) specimens (18 patients) for high-grade dysplasia with adjacent nondysplastic BE (age range, 43–65 years), and 47 nondysplastic biopsy specimens (12 patients) from patients who eventually progressed to dysplasia (BE predysplasia).

#### Cohort 3

This cohort included 31 biopsy specimens from 19 patients with postesophagectomy BE (neo-BE). All patient biopsy specimens were taken a minimum of 2 years after esophagectomy for adenocarcinoma. No data were collected for patient age or BE length. All samples were FFPE.

### Gland Phenotype Identification by Histology and Immunohistochemistry

**Supplementary Figure 1:**
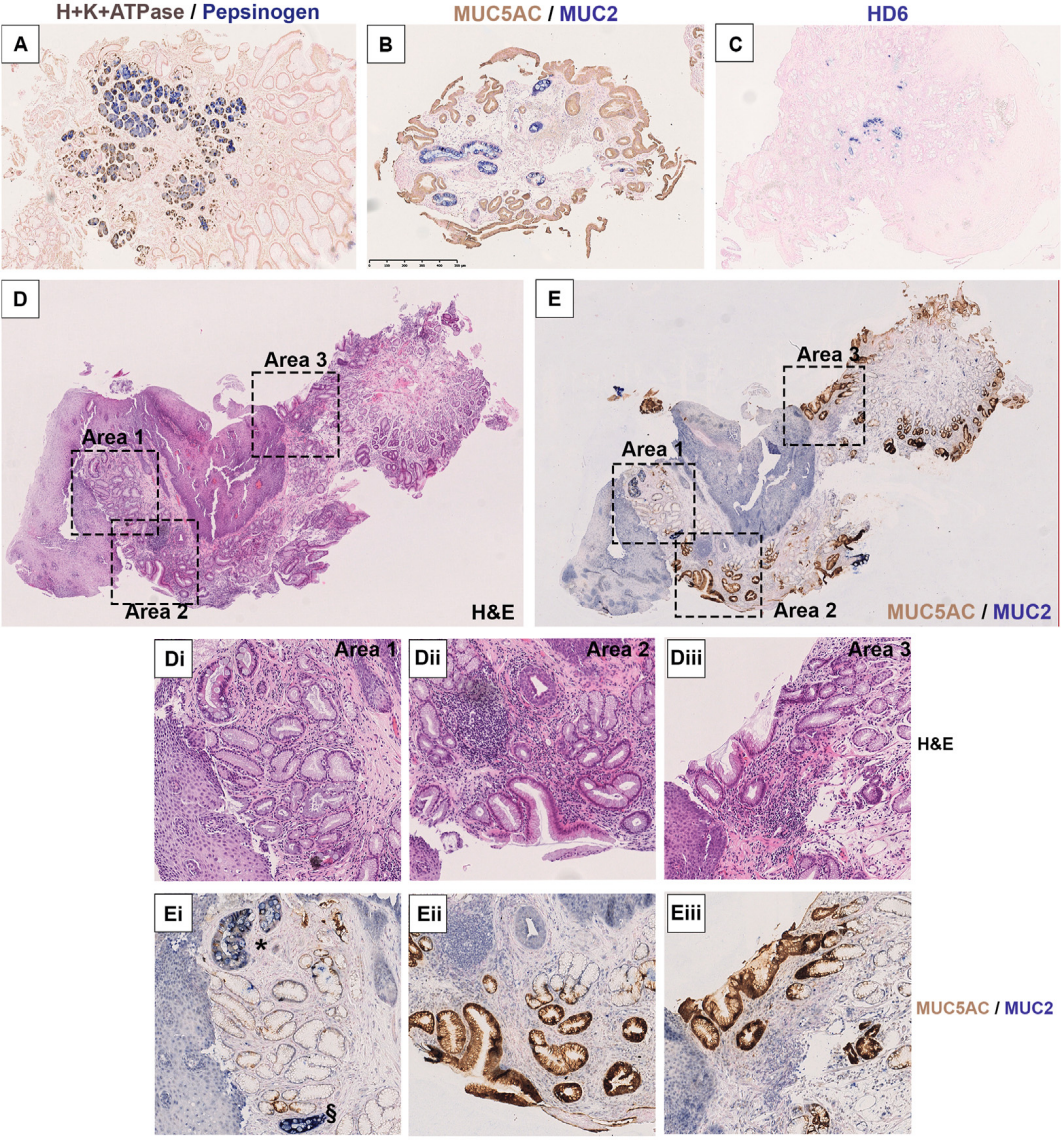
Representative IHC for lineage-identifying antibodies. (*A*) Set 1: H^+^K^+^ATPase (DAB-brown) and pepsinogen (AP-blue). (*B*) Set 2: MUC5AC (DAB-brown) and MUC2 (AP-blue). (*C*) Set 3: HD6 (AP-blue). Additionally, we show that distinct gland types are evident through double IHC. (*D*) An FFPE biopsy specimen that shows both squamous and Barrett’s epithelium stained with H&E and (*E*) a serial section stained with MUC2 and MUC5AC. Three areas are highlighted in higher power (*Di*–*iii*, *Ei*–*iii*). Area 1 shows MUC2^+^ with light MUC5AC^+^ cells within the same specialized gland (∗) and a MUC2^+^MUC5AC^–^ (§) mature intestinal gland. In areas 2 and 3 (*Dii* and *iii*, *Eii* and *iii*) MUC2^+^ cells are completely absent. The IHC clearly defines specialized and cardiac-type epithelium as well as a likely mature intestinal gland. (HD6 staining is not present to confirm the presence of Paneth cells.)

**Supplementary Figure 2:**
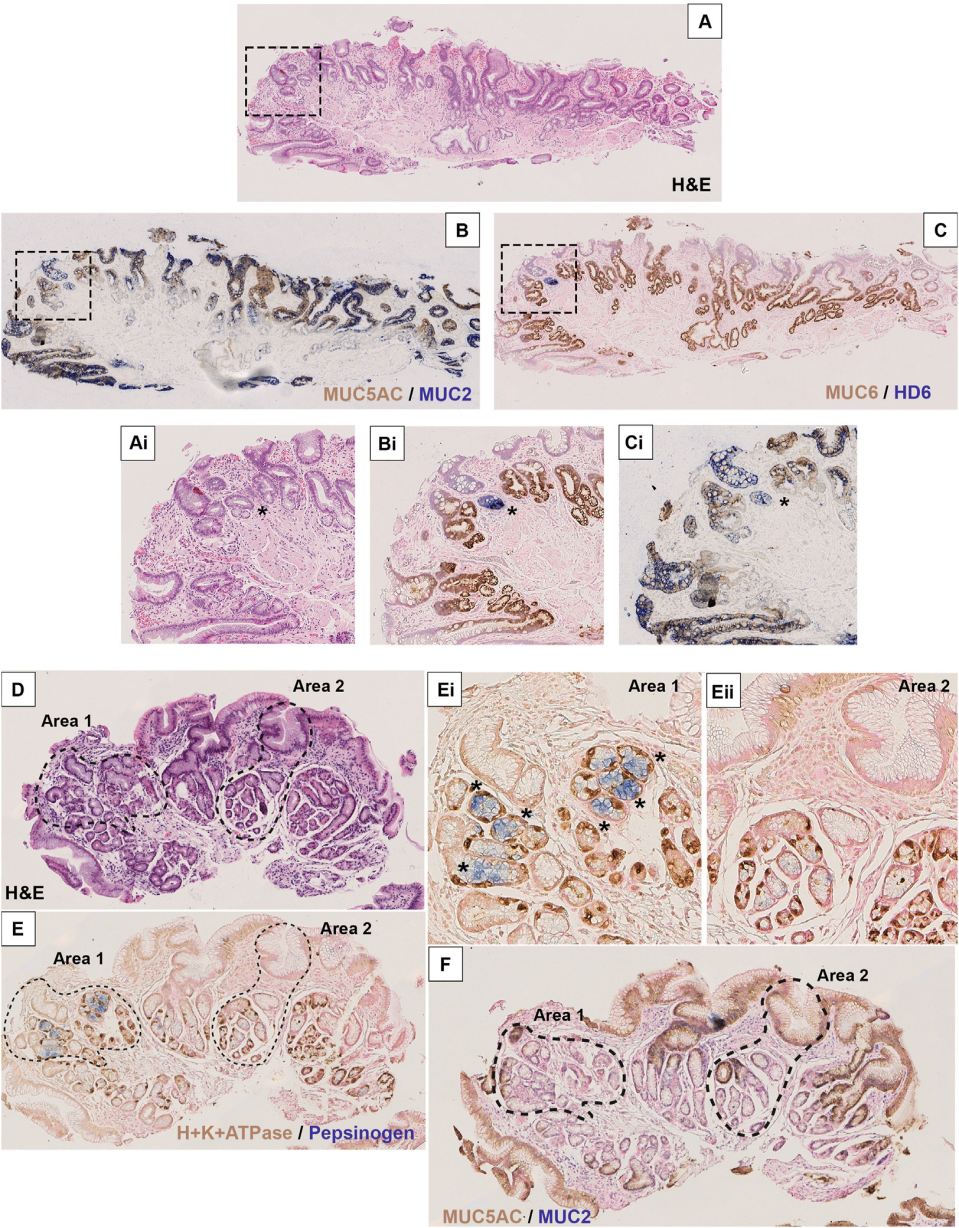
Distinct gland types within biopsy specimens. (*A*) An H&E of a biopsy specimen displaying glandular architecture. (*B*, *C*) Double IHC for MUC2 (AP-blue) + MUC5AC (DAB-brown) and HD6 (AP-blue) + MUC6 (brown), respectively. (*Ai*, *Bi*, *Ci*) Higher-power views of a single mature intestinal gland that is HD6^+^ (∗), where all other glands are specialized, and HD5^–^ with gland bases is evidenced by MUC6 staining. (*D*) A second biopsy specimen shows glandular architecture with a gastric phenotype (H&E). (*E*) Double IHC for H^+^K^+^ATPase (DAB-brown) and pepsinogen (AP-blue) shows 2 areas: area 1 is positive for both markers and is therefore an atrophic corpus phenotype with both parietal and chief cells present (*E* and *Ei*), whereas area 2 only shows positivity for H^+^K^+^ATPase parietal cells (DAB-brown) and is therefore oxyntocardiac (*E* and *Eii*). (*F*) MUC5AC (DAB-brown) + MUC2 (AP-blue) IHC did not show the presence of goblet cells in this biopsy.

Two independent experienced pathologists determined gland phenotype in FFPE and frozen BE tissue sections (M.J. and N.A.W.) by identifying the presence of either chief cells, parietal cells, goblet cells, foveolar cells, or Paneth cells by H&E and by immunohistochemistry (IHC) analysis (Supplementary Figures 1 and 2).

Serial 5-μm FFPE tissue sections were dewaxed in xylene and hydrated through a graded ethanol series to water. Antigen retrieval was performed in boiling Tris-EDTA pH 8.0 (Sigma) or sodium citrate pH 6.0 (FisherChemicals) for 10 minutes depending on each primary antibody (Supplementary Table 3). Endogenous peroxidase activity was blocked with 3% hydrogen peroxide (FisherChemical) for 10 minutes followed by Protein Block, Serum-Free solution (Agilent Technologies Ltd) for 30 minutes. Typically, no endogenous alkaline phosphatase (AP) was detected.

Double IHC was performed in a specific sequence on serial sections of primary antibodies to H^+^K^+^ATPase (horseradish peroxidase [HRP]–3,3′-diaminobenzidine tetra hydrochloride [DAB]) and pepsinogen (AP–blue) (set 1) and for MUC5AC (HRP-DAB) and MUC2 (AP-blue) (set 2) and defensin 5α (HD5, frozen only) or 6α (HD6, FFPE only) (AP-blue) (set 3) (Supplementary Figure 1*A*–*C*, respectively).

Dilutions were performed in Ready-to-Use diluent (Agilent), and primary antibodies were incubated for 1 hour at room temperature, followed by incubation with either a goat anti-mouse IgG (Agilent) or swine anti-rabbit (Sigma) at a 1:200 dilution for 45 minutes at room temperature depending on the primary antibody (Supplementary Table 3). Streptavidin HRP (Agilent) was then added (1:100) and incubated for 30 minutes, and 3,3′-diaminobenzidine tetra hydrochloride peroxidase substrate was added until a brown color developed (Vectro Labs Ltd). This was followed by incubation with a second round of primary antibodies, a secondary-biotinylated antibody, and then a tertiary streptavidin conjugated to AP. Vector blue substrate (Vector Labs Ltd) was then added until a blue color developed. The same protocol was followed for frozen sections with the exception of not performing antigen retrieval, and section thickness was 10 μm.

**Figure 1:**
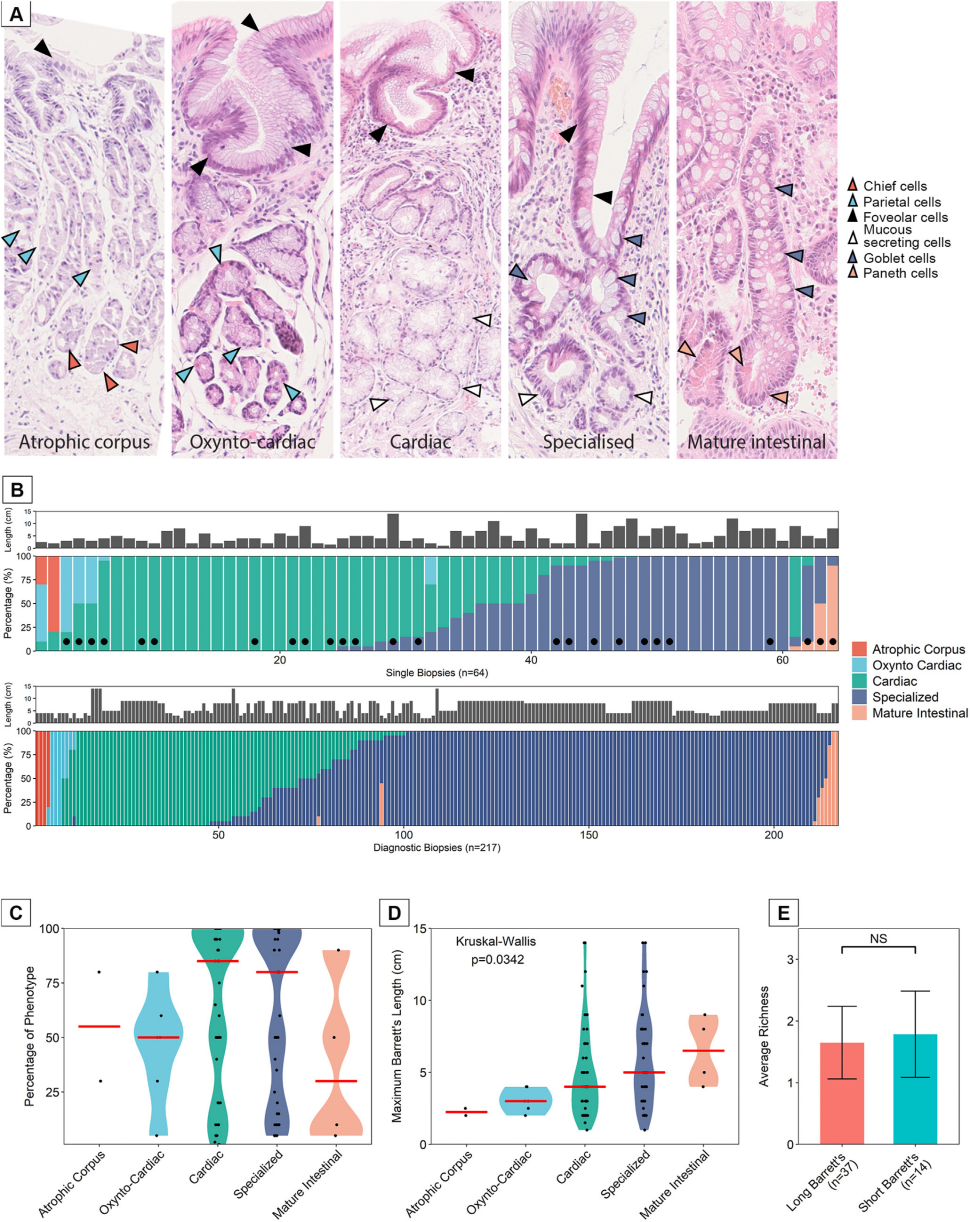
(*A*) The histologic gland phenotypes observed in BE, from left to right: Atrophic corpus glands containing parietal (), chief (), and foveolar cells(); oxyntocardiac glands containing parietal () and foveolar () but not chief cells; cardiac-type glands containing foveolar () and mucous-secreting cells () only; specialized glands containing goblet (), foveolar (), and mucous-secreting cells (); and mature intestinal glands containing goblet () and Paneth cells (). (*B*, *top*) Proportion of gland types within single biopsy specimens, taken from 1.0–2.0 cm proximal of the gastroesophageal junction in observable salmon-pink mucosa. (*B*, *bottom*) Proportion of gland types in biopsy specimens taken throughout the BE lesion from patients marked with • in *B* (*top*). (*C*) A summary of the frequency of gland types through all of the single-biopsy cohort. (*D*) Relationship between specific gland types and the maximum length of the BE lesion. (*E*) The average phenotypic richness within long vs short BE lesions.

A workflow of gland-specific antibodies is shown in Supplementary Method Figure 1. IHC was used to complement H&E analysis, and we show in Supplementary Figure 1*D* and *E* the efficacy of identifying phenotypically distinct glands in a patient whose biopsy contained both MUC2^+^ MUC5AC^+^ (specialized) and MUC2^–^MUC5AC^+^ (cardiac) glands (Supplementary Figure 1*Di*–*iii* and *Ei*–*iii*, respectively). Additionally, we show a similar level of distinction by IHC in mature intestinal MUC2^+^MUC5AC^–^ and specialized glands (Supplementary Figure 2*A*–*Ci*) and the distinction between atrophic corpus and oxyntocardiac glands (Supplementary Figure 2*D*–*F*). The addition of MUC6 to HD6 staining in Supplementary Figure 2*C* was included purely to highlight gland bases.

### Laser-Capture Microdissection

Serial 10-μm frozen sections were cut onto P.A.L.M. membrane slides (Zeiss) previously treated with UV exposure for 30–40 minutes. To delineate gland outline in frozen material, sections were subjected to dual enzyme histochemistry for cytochrome *c* oxidase and succinate dehydrogenase as per previously published protocols.^20^^,^^22^ In all cases, sections were left to dry, and then microdissection was performed using a P.A.L.M laser dissection microscope (Zeiss). Microdissected cells were digested in 14 μL Picopure digestion buffer (Life Technologies) at 65°C for 3 hours, followed by proteinase K inactivation at 95°C for 5 minutes.

### Mitochondrial Polymerase Chain Reaction Sequencing

A nested PCR protocol was used as previously published.^20^ Briefly, the mitochondrial genome from each microdissected area was amplified into 9 2-kilobase fragments, which were subsequently reamplified into 500–base pair fragments. Primer sequences and PCR conditions were used as previously described (Supplementary Table 4).^20^ The second-round PCR primers contained an M13 sequence to facilitate sanger sequencing. PCR products were ExoSaP-treated according to manufacturer’s protocol (GE Healthcare) and Sanger sequenced by Eurofins Genomics. Obtained sequences were viewed using 4Peaks software (https://nucleobite.com) and compared to the revised Cambridge reference sequence using online tools provided at www.mitomap.org/MITOMAP. Polymorphisms and nonepithelial mutations were eliminated from analysis by comparison with sequences from a microdissected area of stroma. Each mutation was confirmed using the same PCR sequencing protocol repeated from the original DNA sample. The mitochondrial DNA (mtDNA) NGS methodology is described in the Supplementary Methods. NGS sequencing data have been deposited at the European Genome-Phenome Archive (EGA), which is hosted by the European Bioinformatics Institute Centre for Genomic Regulation, under accession number EGAS00001005729. Further information about the EGA can be found at https://ega-archive.org.

### Statistics

Statistical analysis was performed using a 1-way analysis of variance (Kruskal-Wallis) test for assessing phenotype distribution. An unpaired Student *t* test or a Mann-Whitney *U* test was used when comparing BE length and diversity, changes in diversity, and changes in phenotype where data were either normally distributed or not, respectively.

Diversity was measured using both a richness score (a record of the number of gland types visible) and the Shannon diversity index^16^ that takes into account the number of gland types and their relative frequency within a tissue specimen. The Shannon diversity index was calculated as

$$\text{Shannon diversity index}=-\overset{s}{\underset{i}{\sum }}p_{i}\;\ln\left(p_{i}\right),$$

where *s* is the number of species, *p*_*i*_ is the frequency of each gland phenotype (*i*) within a tissue specimen. The Shannon diversity index was calculated as the sum of the natural log (ln) of every *p*_*i*_ [ln(*p*_*i*_)]. Significance was determined using a 2-sample Mann-Whitney *U* test.

## Results

### Identification and Distribution of Different Gland Phenotypes in Barrett’s Esophagus

Here, we provide a detailed analysis of gland phenotype from cohort 1 of BE patients (described in the Methods section). Figure 1*A* shows representative H&E FFPE sections of 5 histologically confirmed phenotypes detected in our BE cohort. We additionally developed a lineage-specific expression profile using IHC to assist in identifying gland phenotype (Supplementary Methods Figure 1). Gland species were identified as atrophic corpus (H^+^K^+–^ATPase^+^/pepsinogen^+^), oxyntocardiac (H^+^K^+^ATPase^+^/pepsinogen^–^), simple cardiac type (MUC5AC^+^, MUC2^–^), specialized BE (MUC5AC^+^, MUC2^+^), and mature intestinal (MUC5AC^–^, MUC2^+^, HD6^+^) (Supplementary Figures 1 and 2).

**Supplementary Figure 3:**
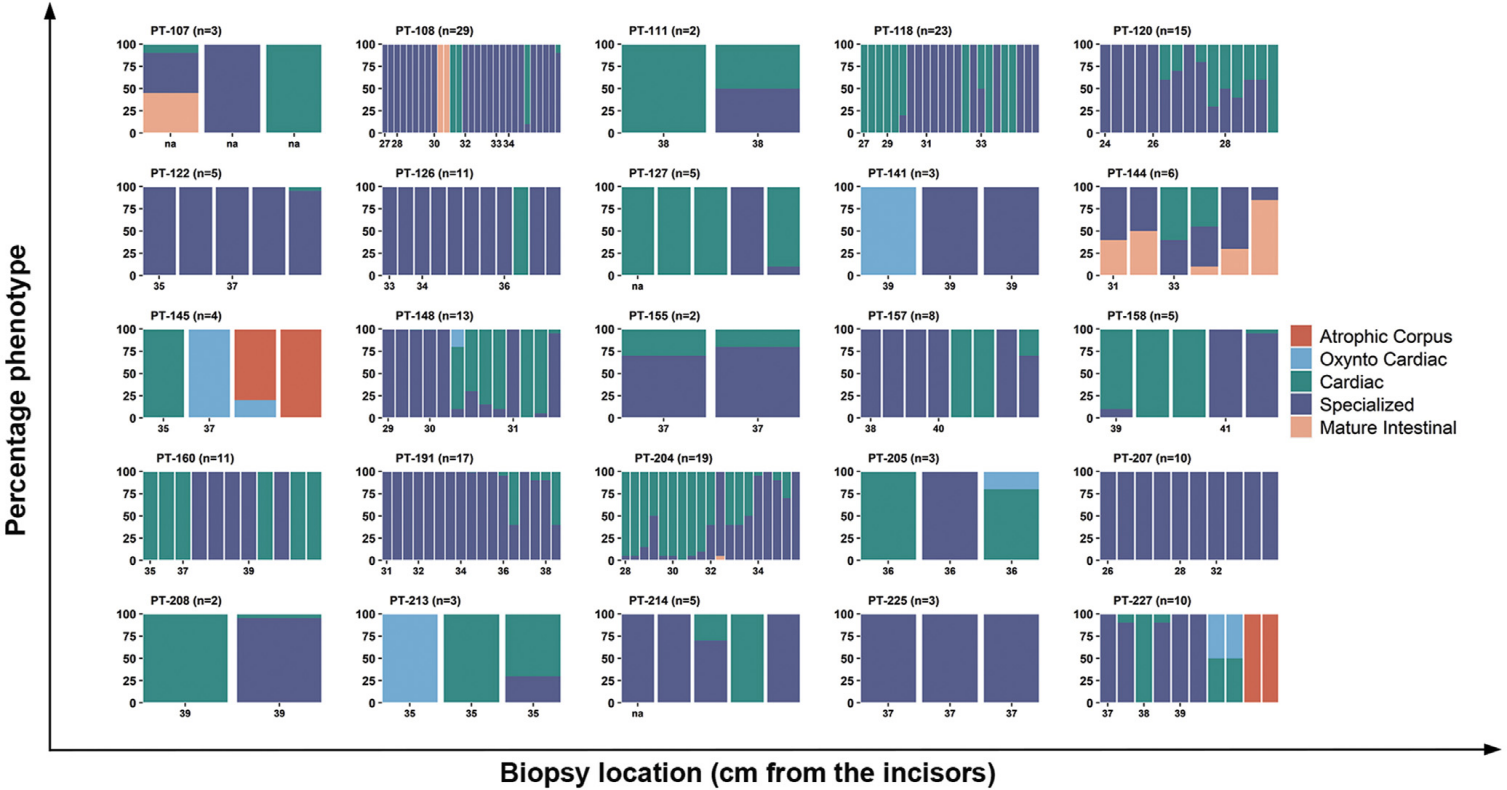
Individual patient distributions of gland phenotype based on available diagnostic tissue blocks. All biopsy specimens from a total of 25 patients, from the cohort described in Figure 1*B* (*top*), taken at the same endoscopy during which the single biopsy specimen was taken, were phenotyped. H&E sections were reviewed by expert pathologists (M.J. and N.A.W.) and 2 experienced researchers (E.C. and S.A.C.M.). Data are presented as percentages of each phenotype within each biopsy specimen at the specific depth in the esophagus at which it was taken.

**Supplementary Figure 4:**
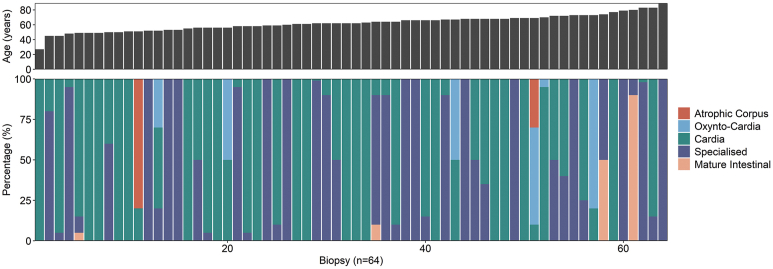
Diversity of BE gland phenotype is not associated with patient age. Each biopsy specimen taken from Figure 1*B* (*top*) was redistributed according to patient age at the time of endoscopy. There is no correlation between patient age and phenotypic diversity.

**Supplementary Figure 5:**
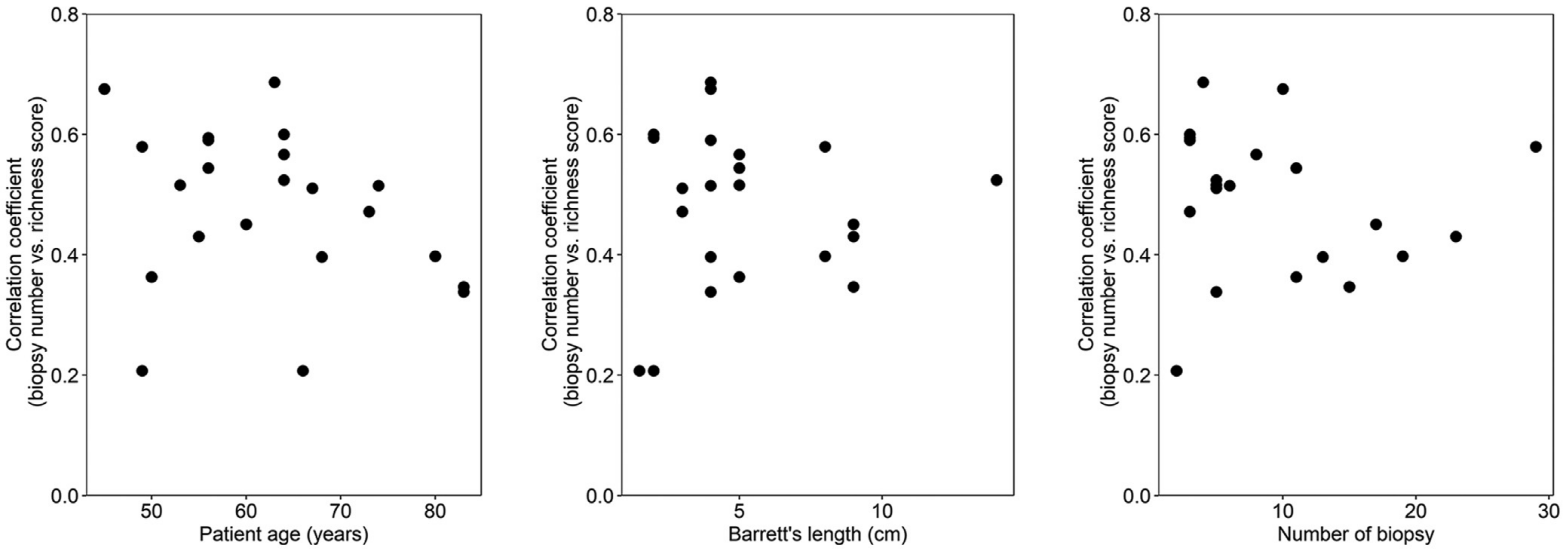
Phenotypic richness in BE is not related to (*A*) patient age, (*B*) lesion size or and (*C*) the number of biopsy specimens taken at endoscopy. The Spearman correlation coefficient was calculated between the number of biopsy specimens sampled and the richness score for each of 1000 bootstrapping replicates for every patient (n = 22). Three patients were removed from this analysis because of unchanging richness across all biopsy specimens.

**Supplementary Figure 6:**
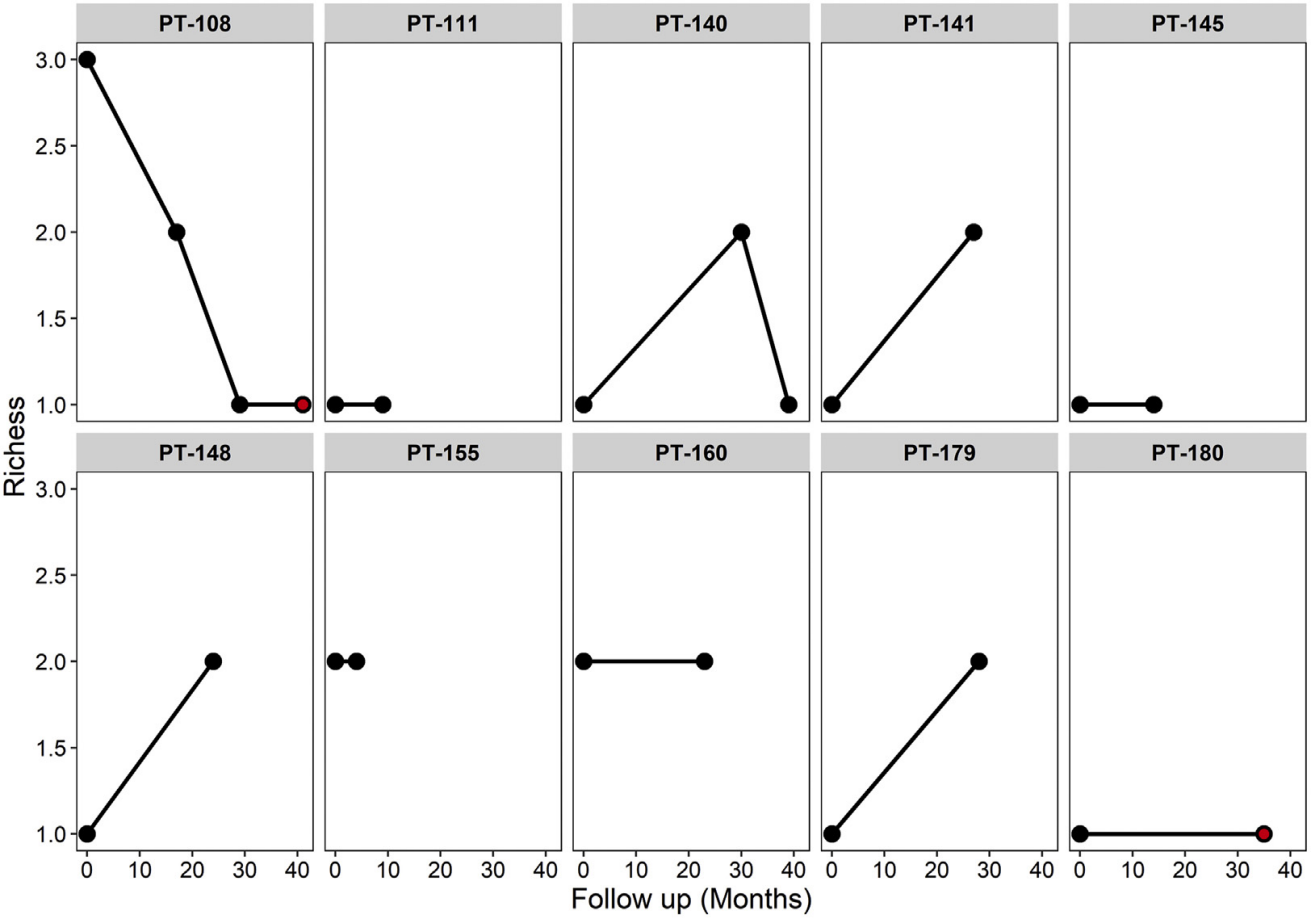
Diversity on follow-up is highly variable. Ten patients with nondysplastic BE showed variable gland richness (number of phenotypes) over time. Five patients showed no change in diversity, 3 showed an increase, 1 showed a decrease, and 1 was variable. A red dot represents a change in phenotype with no change in richness. PT, patient.

Single biopsy specimens (n = 64) were taken at 1.0–2.0 cm above the gastroesophageal junction in a cohort of 51 patients, 10 of whom had follow-up biopsies. The proportion of each gland phenotype within these is shown in Figure 1*B* (*top*), and the corresponding diagnostic biopsy specimens taken during the same endoscopy where available (marked with •) are shown in Figure 1*B* (*bottom*) (n = 25 patients, 217 biopsy specimens). In the single biopsy cohort, 30 of 64 (46.98%) biopsy specimens displayed 1 phenotype, 30 of 64 (46.98%) displayed 2 phenotypes, and 4 of 64 (6.24%) displayed 3. In the small number of cases where 3 phenotypes were observed within single biopsy specimens, these displayed either atrophic corpus or mature intestinal glands. Within the diagnostic biopsy cohort, 149 of 217 (68.67%) displayed one phenotype, 65 of 217 (29.95%) contained 2, and 3 of 217 (1.38%) had 3. Notably, more single phenotypes were observed than in the single biopsy cohort. The individual patient phenotype proportions based on the location of these biopsies within the BE lesion are presented in Supplementary Figure 3. From this, the previously reported distribution of gland phenotype from proximal to distal ends of the lesion^10^ appears to hold, but the distribution of the number of gland phenotypes along this axis was not apparent in the majority of cases. This may be due to cohort differences or overall Barrett segment length. There is a clear dominance of cardiac and specialized gland types from the single biopsy cohort (Figure 1*B* [*top*] and *C*) as well as from the diagnostic biopsy cohort (Figure 1*B* [*bottom*]). There was a positive relationship between the overall size of the lesion and the individual gland types detected at 1.0–2.0 cm, where specialized and mature intestinal glands occur more frequently in the longer rather than the shorter BE segments (Figure 1*D*). This finding is in line with a previous report.^23^ Interestingly, the presence and diversity of specific gland phenotypes from single biopsy specimens at 1.0–2.0 cm was not dependent on BE lesion maximum length (Figure 1*E*), nor was it dependent on patient age (Supplementary Figures 4 and 5). Phenotypic richness was not associated with patient segment length or the number of biopsy samples taken at any single endoscopy as per bootstrapping analysis (Supplementary Figure 5). Additionally, 10 patients had follow-up over time data available, 5 of which (50%) showed no change in diversity (Supplementary Figure 6).

### Mitochondrial DNA Mutations in Barrett’s Esophagus, Anatomic Gastric Cardia, and Squamous Tissue

**Supplementary Figure 7:**
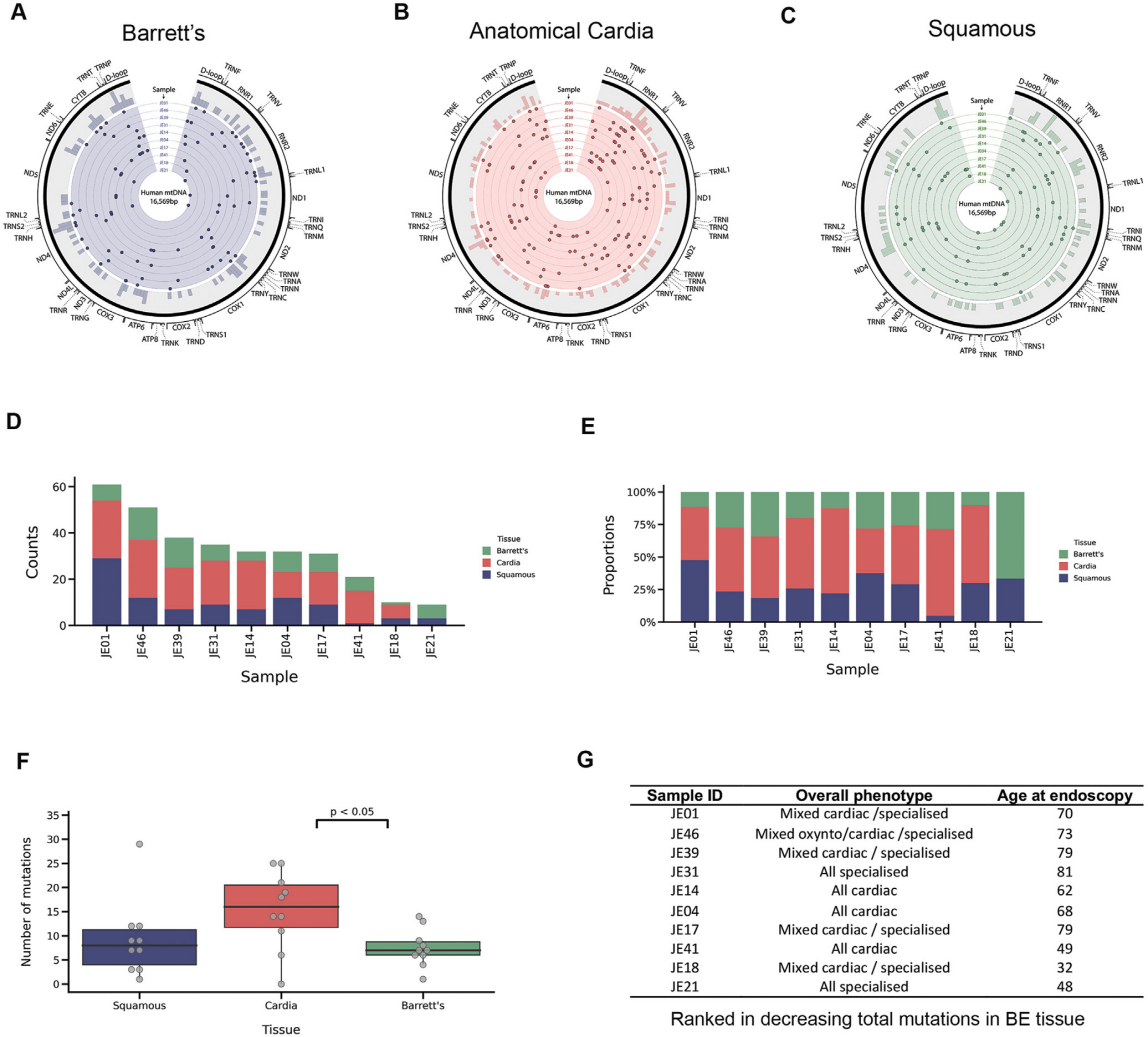
NGS of BE, gastric cardia, and adjacent squamous mtDNA from matched patients. (*A*–*C*) Circos plots showing the distribution of all somatic variants in (*A*) Barrett’s epithelium, (*B*) anatomic gastric cardia epithelium, and (*C*) squamous. n = 10 for each. (*D*, *E*) Mutation burden as per (*D*) normalized mutation count for each tissue type and (*E*) the proportion of mtDNA mutations in each tissue for each patient. (*F*) A comparison of the number of variants observed in each tissue. A significantly greater number of mutations was observed in the cardia compared with BE (*P* < .05). (*G*) Mutation burden was not associated with gland phenotype. Patients are ordered by decreasing number of mutations, and a trend was observed in mutation frequency with increasing age (Pearson’s correlation *R*^2^ = 0.72, *P* = .02).

To determine the concentration and distribution of somatic mtDNA mutations, we performed NGS on laser-capture–microdissected epithelium from frozen sections of biopsy specimens taken from 10 patients with matched BE, anatomic gastric cardia, and squamous samples (Supplementary Materials and Methods). Single-gland sequencing was not possible because of the concentration of mtDNA being below the sensitivity of the NGS assay. Mutations were found in all samples except the gastric cardia of 1 patient and showed no significant hotspots within the mitochondrial genome of any patient (Supplementary Figure 7*A*–*C*). A similar concentration of BE mtDNA mutations was detected compared to other reports.^24^ A variable mutation count (Supplementary Figure 7*D*) and proportion of total mutations of each tissue type in each patient (Supplementary Figure 7E) was observed. There were significantly more mutations in the gastric cardia compared to matched BE (Supplementary Figure 7*F*). No shared somatic mtDNA mutations were observed between any patient samples. Furthermore, there were no significant differences between any BE phenotype and mutation numbers; however, there was a trend with younger patients displaying the fewest mtDNA mutations (Pearson correlation *R*^2^ = 0.72, *P* = .02) (Supplementary Figure 7*G*). This fits with previous studies showing that somatic mtDNA mutations are age dependent.^25^ These data show the presence of frequent mtDNA mutations in BE independent of gland phenotype.

### Evolution of Gland Phenotypes in Barrett’s Esophagus

**Figure 2:**
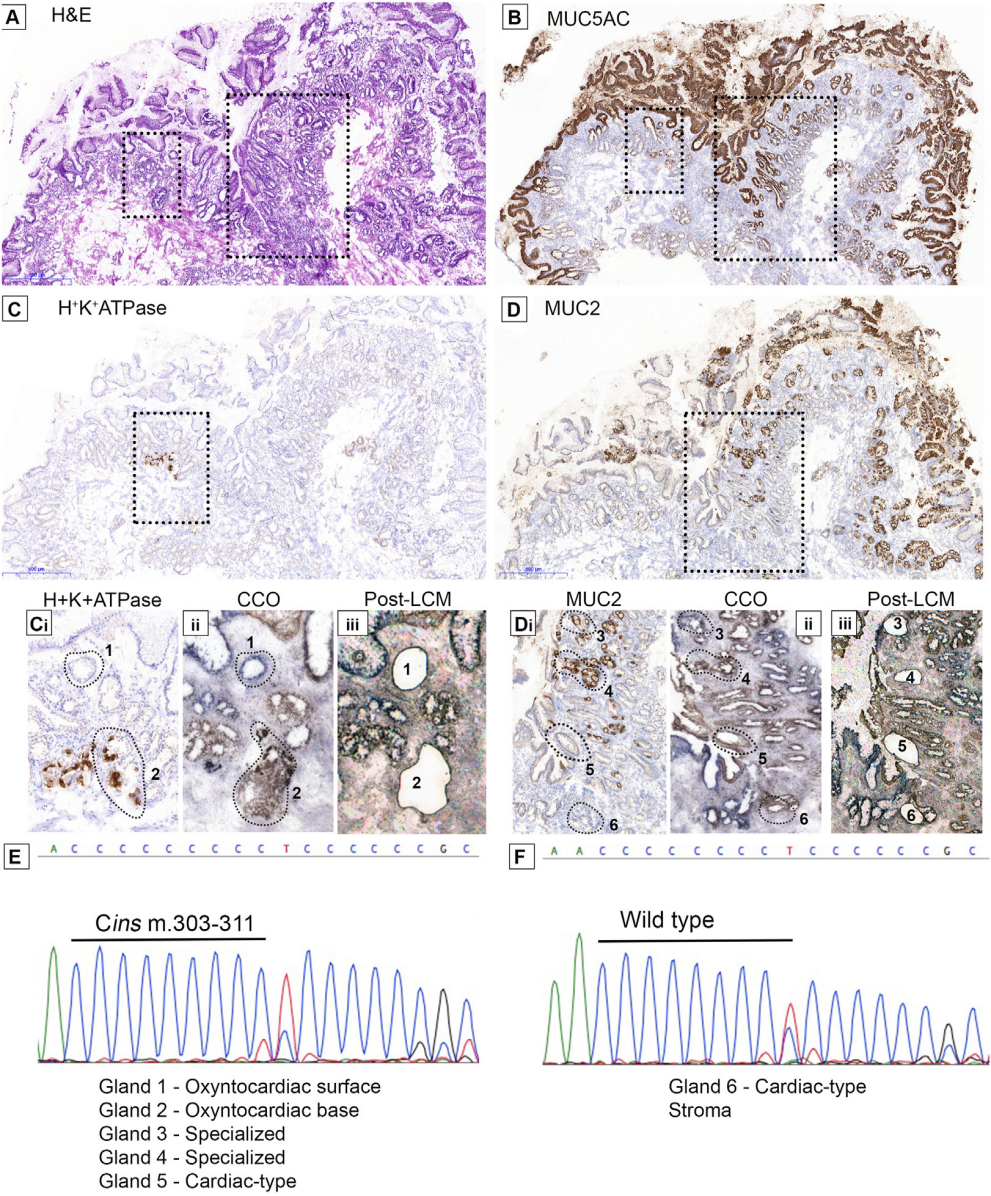
A common ancestry between oxyntocardiac, cardiac-type, and specialized epithelium in BE. (*A*) H&E with areas of interest outlined. (*B*) MUC5AC is extensively expressed on all surface foveolar cells. (C) H^+^K^+^ATPase^+^ parietal cell–containing glands and (*D*) MUC2^+^ goblet cell glands on the same section. (*Ci*–*iii*) and (*Di*–*iii*) show high-power magnification images of *C* and *D*, respectively, with adjacent cytochrome *c* oxidase staining (*Cii*, *Dii*) and postmicrodissection images (*Ciii*, *Diii*). (*E*) PCR sequencing showed a common, complex *m.303-311 Cins* mutation in the *MT-OHR* region of the mitochondrial genome present in oxyntocardiac MUC5^+^MUC2^–^ surface (*1*) and H^+^K^+^ATPase^+^ base (*2*) glands as well as MUC5AC^+^MUC2^+^ specialized glands (*3* and *4*) and a MUC5AC^+^MUC2^–^ cardiac-type gland (*5*). (*F*) The stroma and 1 other cardiac-type MUC5AC^+^MUC2^–^ gland (*6*) sequenced were wild type. All microdissected glands are traced by dotted outlines.

These analyses underscore significant phenotypic heterogeneity, even in biopsy specimens from uncomplicated Barrett segments. Multiple phenotypes in a single biopsy may be a consequence of independent parallel evolution or, alternatively, shared branching evolution. To investigate phenotypic gland evolution in BE, we determined if divergent gland phenotypes within biopsy specimens share a common ancestor. In total, 10 snap-frozen biopsy specimens from cohort 1b that showed at least 2 gland types, as verified by IHC, were subjected to laser capture microdissection mtDNA Sanger sequencing. Glands of each individual phenotype were microdissected. Of these cases, 6 did not show any mtDNA mutations, and 1 demonstrated a mutation that was shared by nonepithelial cells (and was excluded). Of the remaining 3 cases, 1 case (H&E [Figure 2*A*] and MUC5AC [Figure 2*B*]) showed the presence of both H^+^K^+^-ATPase^+^ parietal cell–containing oxyntocardiac glands (Figure 2*C* and *Ci* at high power), MUC5AC^+^MUC2^+^ goblet cell-containing specialized glands, and MUC5AC^+^MUC2^–^ cardiac-type glands (Figure 2*B*, *D*, and *Di* at high power). Cytochrome *c* oxidase staining on an adjacent slide was shown as pre-LCM (Figure 2*Cii* and *Dii*), and post-LCM micrographs are shown (Figure 2*Ciii* and *Diii*). A shared homoplasmic somatic *m.303-311 Cins* mtDNA mutation in the H-strand replication origin region (*MT-OHR*) (Figure 2*E* and *F*) was observed between the entire parietal cell-rich area (glands 1 [surface] and 2 [base]), 2 MUC5AC^+^MUC2^+^ specialized glands (glands 3 and 4), and a single cardiac-type MUC5AC^+^MUC2^–^ gland (gland 5). This mutation was not present in a nearby MUC5AC^+^MUC2^–^ gland (gland 6) and stroma. This indicates the presence of clonal lineages within multiple gland phenotypes and shows, for the first time to our knowledge, a clonal relationship between gastric-like glands (oxyntocardiac and cardiac) and specialized glands in BE.

**Figure 3:**
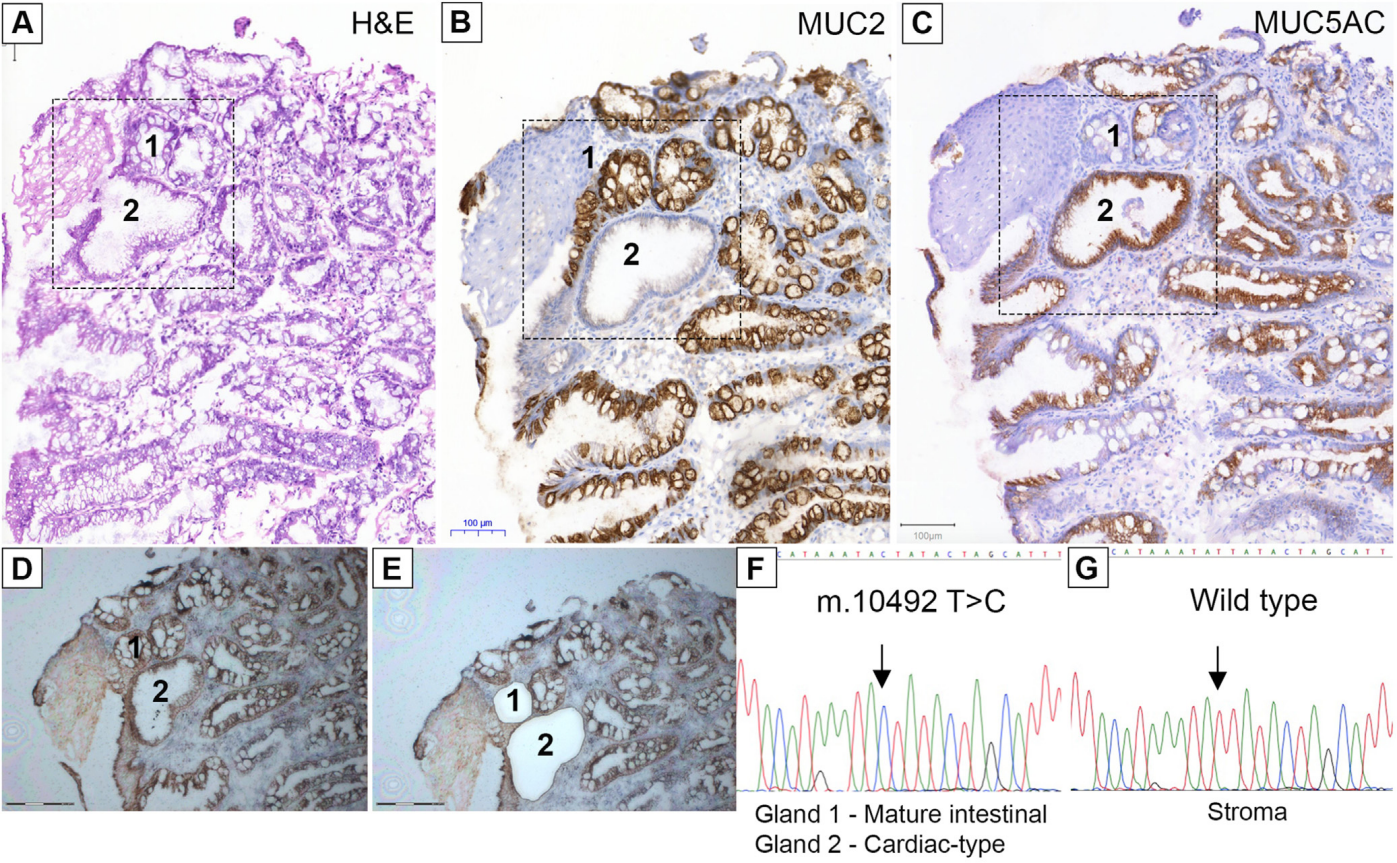
A common ancestry between cardiac-type and mature intestinal epithelium. (*A*) H&E of a BE biopsy specimen containing both cardiac and mature intestinal epithelium (outlined region). (*B*) MUC2^+^ and MUC2^–^ and (*C*) MUC5AC^+^, MUC5AC^lo^, and MUC5AC^–^ glands are present. (*D*, *E*) Pre- and post-LCM images, respectively. Gland 1 is a MUC2^+^MUC5AC^–^ mature intestinal gland. Gland 2 is a MUC2^–^MUC5AC^+^ cardiac-type gland. (*F*, *G*) Glands 1 and 2 showed a common *m.10492T>C* mutation in the *MT-ND4L* region of the mitochondrial genome not present in stroma and other glands.

Furthermore, we investigated an additional frozen biopsy specimen (H&E) (Figure 3*A*) wherein all glands bar a single gland were MUC2^+^ (Figure 3*B*). MUC5AC (Figure 3*C*) staining showed a mixture of positive and negative glands, indicating the presence of cardiac-type, specialized, and mature intestinal glands. LCM of the single MUC2^–^MUC5AC^+^ cardiac-type gland and a neighboring MUC2^+^MUC5AC^–^ mature intestinal gland (cytochrome *c* oxidase-stained before and after LCM) (Figure 3*D* and *E*) showed a shared homoplasmic *m.10492 T>C* mutation in the *MT-ND4L* region (Figure 3*F*) that was not present elsewhere in the biopsy specimen (Figure 3*G*).

**Figure 4:**
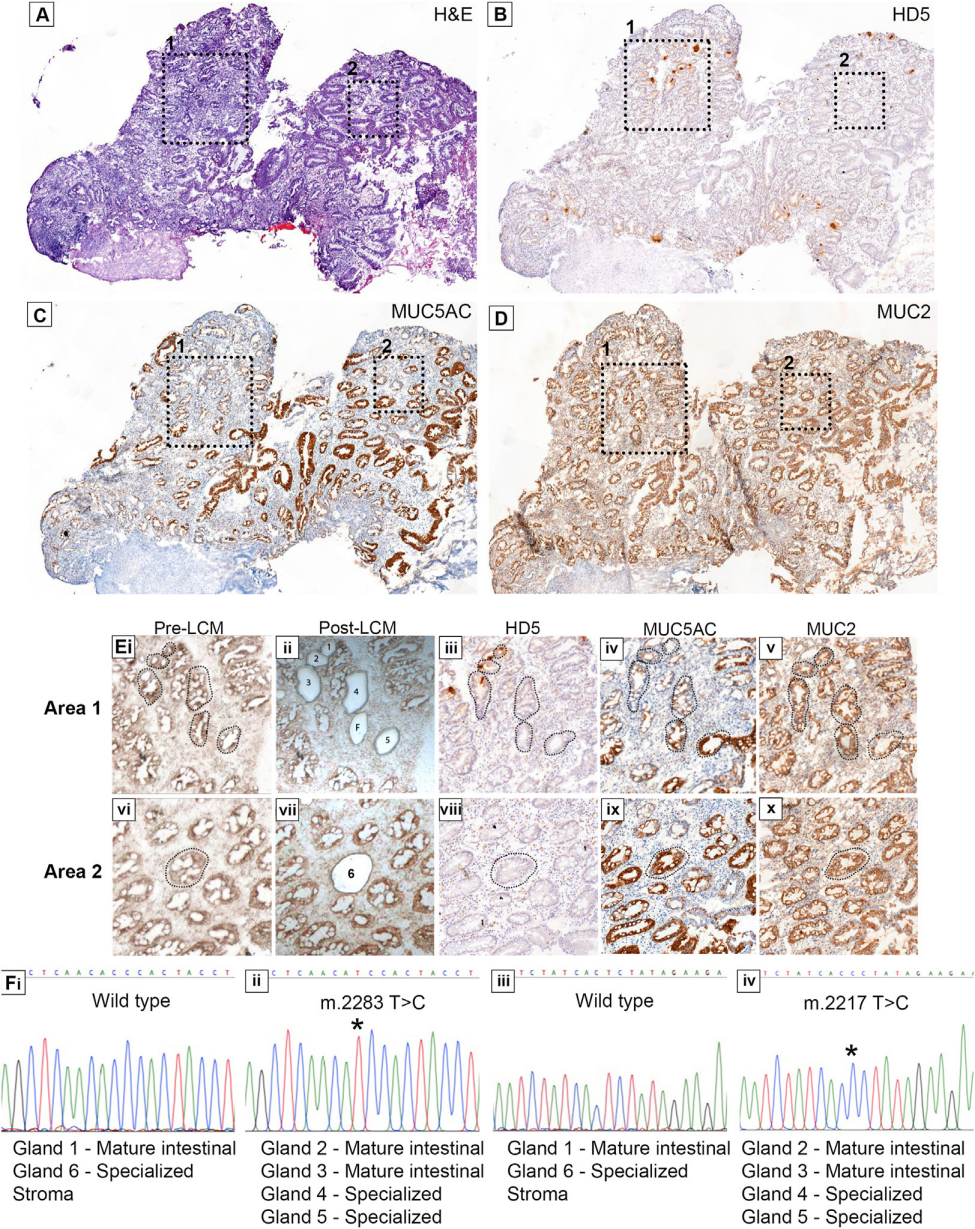
A common ancestry of specialized and mature intestinal glands in BE. (*A*) H&E of a biopsy containing both gland phenotypes confirmed by IHC for (*B*) HD5^+^ and HD5^–^ glands, (*C*) MUC5AC^+/lo^ and MUC5AC^–^ glands, and (*D*) MUC2^+^ glands. Dash-lined boxes indicate 2 areas of interest. (*Ei*–*x*) Show the 2 areas of interest at higher power, with pre- and post-LCM images (dotted outlines) as well as mucin and defensin staining of each area. Gland marked *F* (*Eii*) failed amplification. (*Fi*–*iv*) Two somatic mtDNA mutations (*m.2283 T>C* and *m.2217T>C*) in HD5^+^MUC5AC^–^MUC2^+^ mature intestinal glands (2 and 3) and in 2 specialized glands; 1 being HD5^–^MUC5AC^lo^MUC2^+^ and the other HD5^–^MUC5AC^+^MUC2^+^ (glands 4 and 5, respectively). The variants were not present in a neighboring HD5^+^MUC5AC^–^MUC2^+^ mature intestinal gland (1) and a distant HD5^–^MUC5AC^+^MUC2^+^ specialized gland (6) and the surrounding stroma. Mutations are marked as ∗.

In a third patient, we observed the presence of specialized (HD5^–^MUC5AC^+ or lo^MUC2^+^) and mature intestinal (HD5^+^MUC5AC^–^MUC2^+^) glands (Figure 4*A* [H&E], *B* [HD5], *C* [MUC5AC], and *D* [MUC2]) and with high-power images (Figure 4*Ei*–*x*). We microdissected 7 glands in total (3 HD5^+^, 4 HD5^–^). We detected 2 homoplasmic somatic mtDNA mutations (*m.2283 T>C* and *m.2217 T>C*), both located in the MT-RNR2 region (Figure 4*Fi*–*iv*) in 2 mature intestinal HD5^+^MUC5AC^–^MUC2^+^ glands (glands 2 and 3) (Figure 4*Eiii*–*v*). Additionally, 1 HD5^–^MUC5AC^lo^MUC2^+^ and 1 HD5^–^MUC5AC^+^MUC2^+^ gland (both considered specialized) also contained both variants (Figure 4*Eiii*–*v*). However, a neighboring mature intestinal HD5^+^MUC5AC^–^MUC2^+^ and a distant specialized HD5^–^MUC5AC^+^MUC2^+^ gland (glands 1 and 6, respectively) were both wild type for both variants (Figure *Eiii*–*v* and *viii*–*x*). These data show that mature intestinal and specialized glands can share a common ancestor. Furthermore, this provides additional evidence of a multiclonal landscape in BE. Taking all 3 patients together, these data show shared common ancestry between phenotypically distinct glands in BE, suggesting an evolutionary process that accounts for the presence of phenotypic diversity.

### Phenotypic Diversity in Nondysplastic Barrett’s Esophagus Adjacent to Dysplasia

Overall, these data show the diversity and clonal relationship of gland phenotype evolution in nondysplastic BE. Our analysis suggests that phenotypic transitions are bottleneck events. Successive bottlenecks increase the risk of progression. Therefore, we hypothesized that BE associated with dysplasia would show greater evidence of phenotypic diversity. To determine if gland phenotype is altered in BE progression to dysplasia or cancer, we investigated an additional group of patients (cohort 2; see Methods section) that included 19 patients who had no history of dysplasia or cancer (99 biopsy specimens), 12 patients (47 biopsy specimens) with nondysplastic BE taken before a diagnosis of dysplasia (predysplastic BE), 18 patients (33 EMR specimens) with confirmed dysplasia or cancer who also displayed surrounding nondysplastic BE, and 19 patients (31 biopsy specimens) who developed nondysplastic postesophagectomy BE (cohort 3, neo-BE) within 2 years of removal of the lower esophagus.

**Figure 5:**
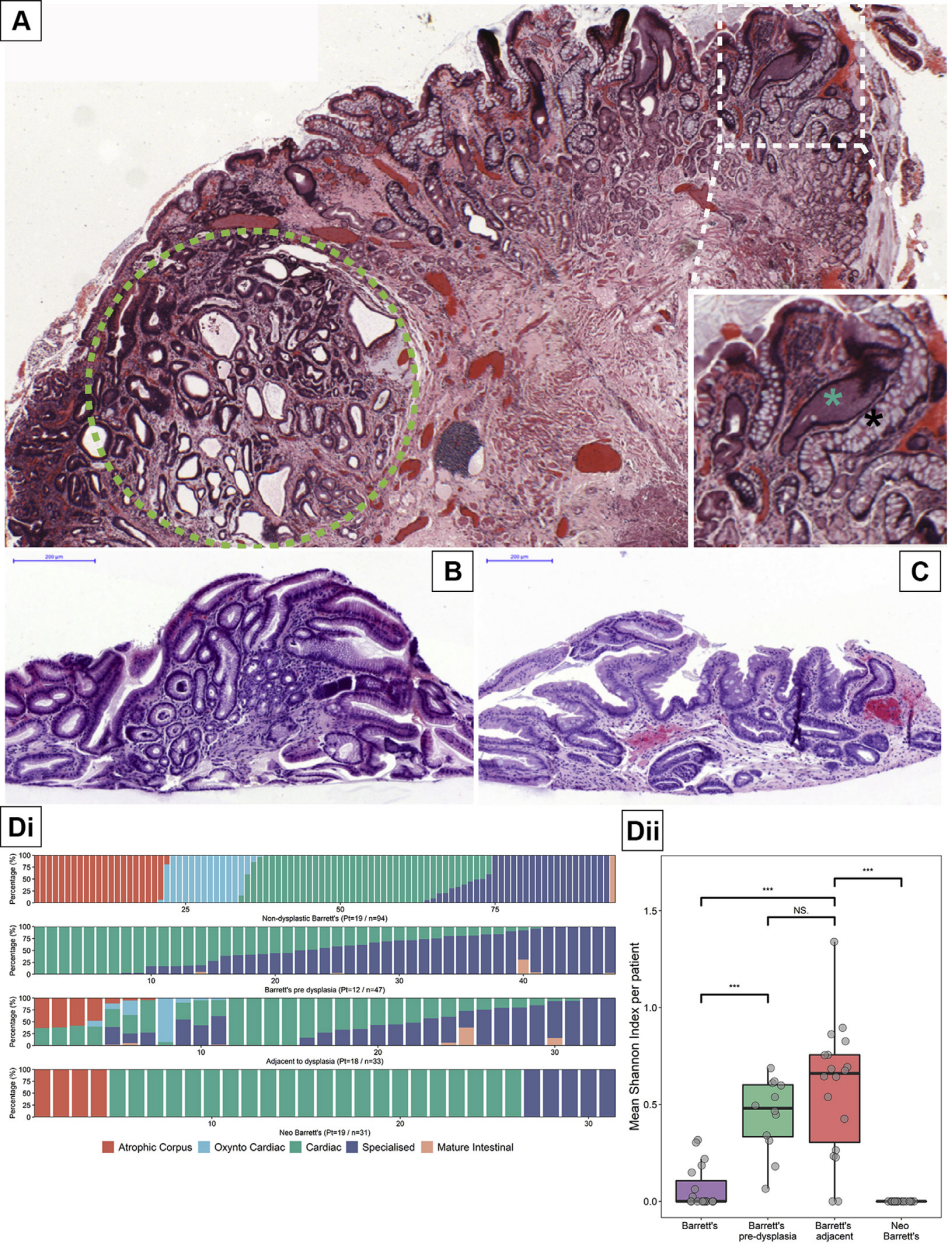
BE gland phenotype diversity is associated with dysplasia. (*A*) An endoscopic mucosal resection H&E showing an area of high-grade dysplasia (green dashed circle) with surrounding nondysplastic BE showing the presence of multiple gland phenotypes (white dashed box). The inset shows a distinct cardiac-type gland () and a mature intestinal gland (∗) adjacent to each other. (*B*) and (*C*) display postesophagectomy BE (neo-BE) biopsy specimens showing cardiac-type epithelium and specialized epithelium, respectively. (*Di*) The percentage of each phenotype observed in 94 biopsy specimens (from 19 patients) with BE and no history of dysplasia, 47 biopsy specimens (from 12 patients) with nondysplastic BE before developing dysplasia, 33 specimens of EMRs (from 18 patients) with BE adjacent to dysplasia, and 31 neo-BE biopsy specimens (from 19 patients). (*Dii*) The mean Shannon diversity index per patient for each patient set from *Di* (∗∗∗ = *P* < 0.001, NS = not significant).

**Supplementary Figure 8:**
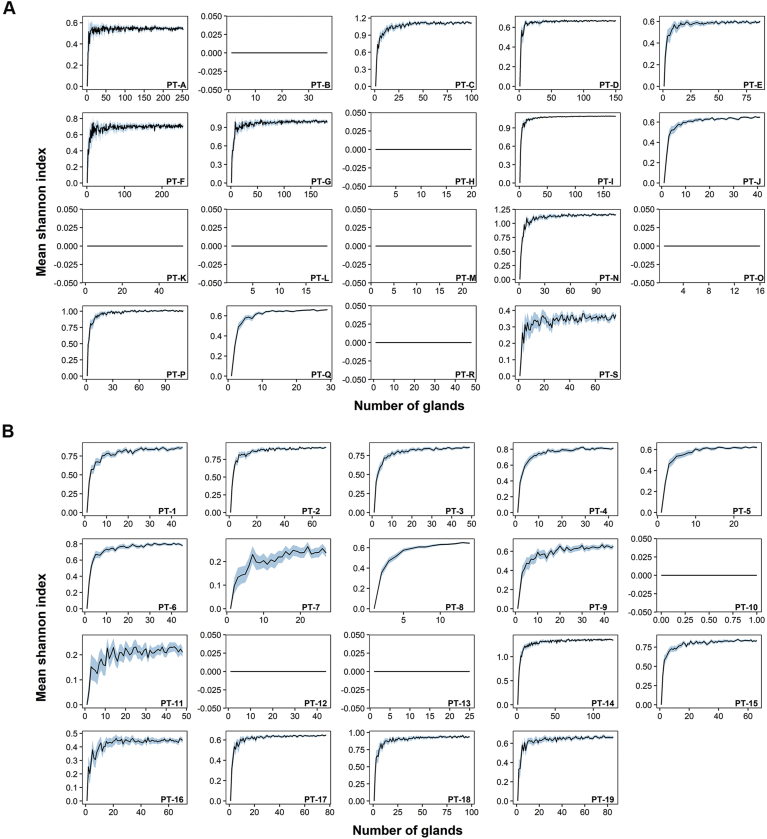
The number of glands sampled in the BE and BE adjacent to dysplasia cohorts was sufficient to provide a reliable Shannon diversity index result. Each graph represents an individual patient. The blue shaded area represents the 95% confidence interval. The Shannon diversity index is derived from a bootstrapping subset (n = 5000) from either the BE or the BE adjacent to dysplasia cohort. In the vast majority of cases, a stable Shannon diversity index was achieved in the simulation below or at the number of glands used in the experimental data set. Neo-BE was not included because no diversity was observed. PT, patient.

Figure 5*A* shows a representative H&E section of an endoscopic resection specimen showing nondysplastic BE (*white dashed square*) adjacent to BE neoplasia (intramucosal adenocarcinoma, *green dashed circle*). It is clear from this section that there are multiple gland phenotypes in the nondysplastic BE adjacent to BE neoplasia and at high power magnification, it is clear that these are distinct glands (Figure 5*A*, *inset*). Representative H&E images from patients with neo-BE are shown in Figure 5*B* and *C*. We then compared the phenotypic proportions of BE, BE predysplasia, BE adjacent to dysplasia, and neo-BE using a biopsy-by-biopsy approach (Figure 5*Di*). We calculated the Shannon diversity index (see Methods section) based on the percentage of glands of a given phenotype within each specimen and within each of the 4 groups (Figure 5*Dii*). Interestingly, we find that BE predysplasia and BE adjacent to dysplasia is significantly more phenotypically diverse compared to BE biopsy specimens from patients without dysplasia or those with neo-BE (Figure 5*Dii*). Individual biopsy specimens capture a smaller mucosal surface area compared to shoulder regions of EMR specimens, which may underestimate diversity in the biopsy group. To address this, bootstrapping analysis was performed on all the biopsy specimens in our cohort (Supplementary Materials and Methods), which showed that randomly sampling a larger number of glands from these biopsy specimens would not have altered the outcome of the Shannon diversity index (Supplementary Figure 8).

**Supplementary Figure 9:**
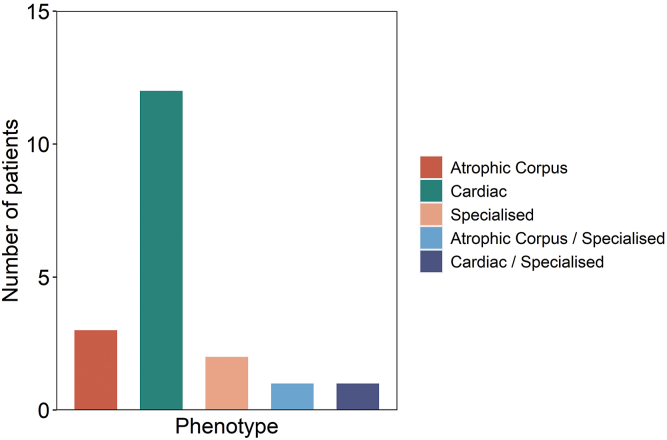
Gland phenotype in neo-BE cases. Thirty-one biopsy specimens from 19 neo-B patient biopsies were phenotyped. The majority (n = 22) of patient biopsy specimens showed a pure cardiac-type phenotype.

**Supplementary Figure 10:**
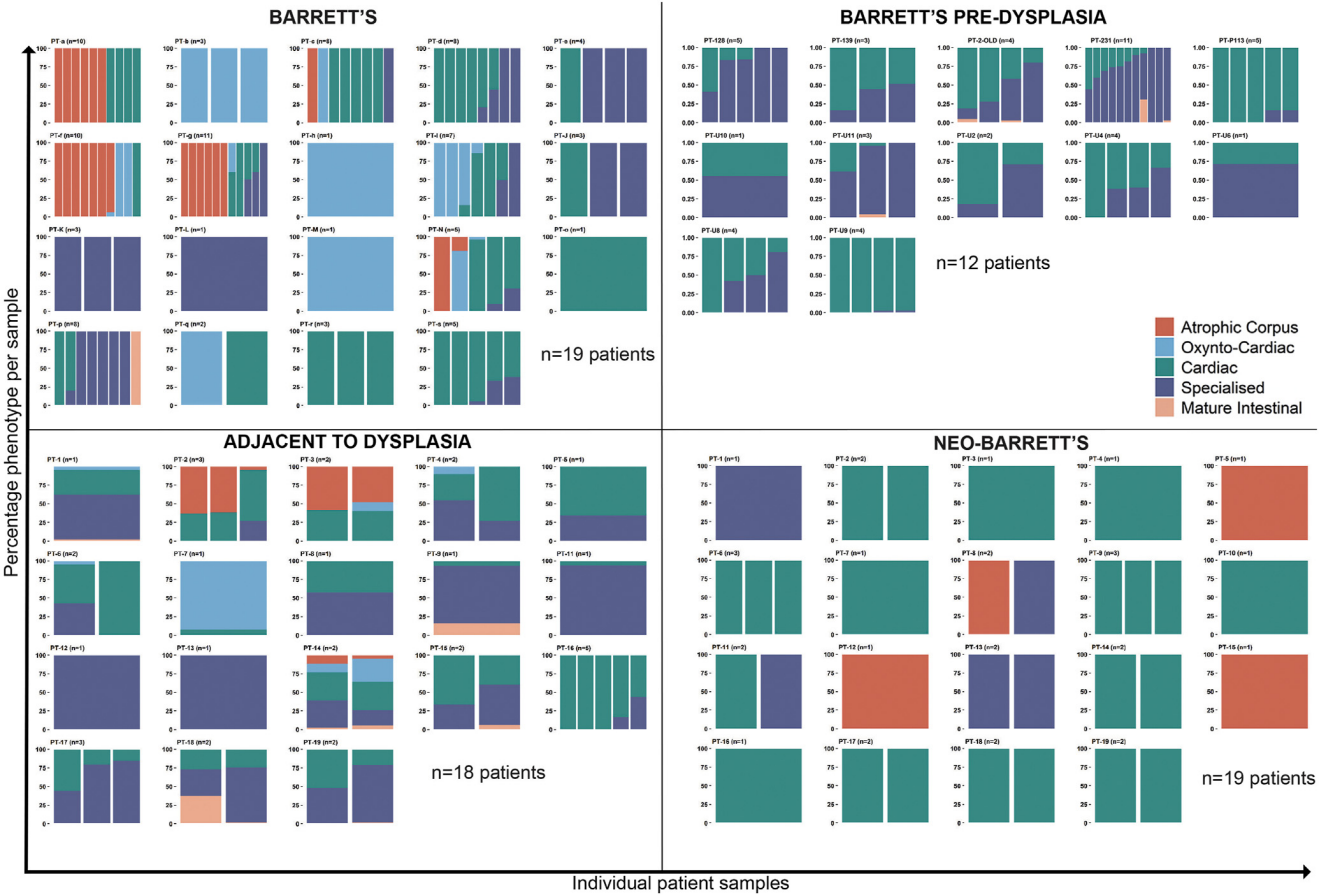
A per-patient analysis of observed gland phenotypes. Phenotypic diversity within nondysplastic BE (*top left*), predysplastic BE (*top right*) adjacent to dysplasia, BE (*bottom left*), and neo-BE (*bottom right*). Each column represents a single biopsy taken at a known site within the esophagus.

Neo-BE biopsy specimens showed no per-biopsy diversity, and the dominant phenotype observed was cardiac (n = 22/31 biopsy specimens in 13/19 patients), as has been observed before.^26^ Specialized and atrophic corpus-type glands were identified in a minority of patients (n = 5/31 biopsy specimens in 4/19 patients and n = 4/31 biopsy specimens in 4/19 patients, respectively). Two neo-BE patients showed more than 1 phenotype (Supplementary Figure 9) but in distinct biopsy specimens only. A patient-per-patient biopsy phenotype distribution for all 4 groups is shown in Supplementary Figure 10. These data show that areas of nondysplastic BE adjacent to dysplasia and BE predysplasia are significantly more diverse than BE or neo-BE, indicating that diversity develops before the onset of dysplasia. This suggests that phenotypic diversity could act as a potential predictive biomarker for progression in surveillance biopsies.

## Discussion

The study of gland phenotype in BE has been largely overlooked.^10^, ^11^, ^12^ Considering that all BE diagnoses are based on phenotypic observations, there is an unmet need to determine the range, evolution, mechanism, and diversity of these phenotypes. Our cohorts appear to reflect those included in previous studies by identifying similar gland types,^10^ but with a much broader interpretation of phenotypes and their diversity. The ancestral relationships we uncover between gland phenotypes provide important information about the evolution of BE to cancer. We show that evolution of BE can involve a change of phenotype at the stem cell level (niche succession^27^^,^^28^). Importantly, we show that a mature gastric gland phenotype expressing parietal cells shares a common ancestor with specialized BE, indicating a phenotypic adaptation of BE along a gastric differentiation, presumably as a result of natural selection acting on gastric phenotypes. We show that multiple phenotypic changes represent clonal phenotypic evolution within BE and that an increase in their diversity is associated with progression. However, de novo postesophagectomy BE does not show any within-biopsy diversity, and this suggests that gland phenotype diversity is acquired over time and may not be present during the early development of BE.

We observe 5 well-defined gland phenotypes in our cohort of biopsy specimens taken at the same anatomic location within the esophagus (1.0–2.0 cm proximal of the gastric folds). Of note, we did not observe any pancreatic metaplasia in any of our cohorts.^29^ Previous reports have suggested that there is a distribution of gland type within the length of the BE segment.^10^ Although we observe a relationship between the proportions of individual gland types present at 1.0–2.0 cm and the overall size of the BE lesion, diversity of gland type is not dependent on lesion size, and cases where more than 2 phenotypes are present in the same biopsy specimen are rare. This is confirmed when we investigate all biopsy specimens taken at each particular endoscopy and observe no change in gland phenotype diversity between the single biopsy set (Figure 1*B*, *top*) compared with the diagnostic biopsy set (Figure 1*B*, *bottom*). The distribution of gland phenotype is likely to be determined by the surrounding microenvironment.

Here, we report that gland phenotype can evolve and show that distinct gland types, each with a specific combination of differentiated epithelial cells, can share common ancestry. In particular, we show that cardiac-type and mature intestinal glands in direct proximity to each other have a common mtDNA mutation that can only be explained by the mutation arising in an ancestral gland and being passed to its daughter glands as it divides by gland fission.^30^ The odds of 2 glands with distinct epithelial phenotypes possessing the same homoplasmic or highly heteroplasmic mtDNA mutation independently is vanishingly small.^20^ It is, therefore, highly likely that gland fission is the mechanism of clonal expansion within the metaplastic esophagus as it is in the normal, unperturbed gastrointestinal tract.^20^ Importantly, we observed a shared somatic mtDNA mutation between gastric-type glands (oxyntocardiac and cardiac) and specialized glands, showing that intestinal and mature gastric lineages can share ancestry. Although glandular differentiation within the BE segment is traditionally depicted as one of increasing intestinalization per se, these data show that metaplastic glands can also follow a gastric line of differentiation (Figure 2). Individual patient characteristics such as smoking, obesity, or age may influence gland phenotype selection. Although we do not have sufficient cases to infer a relationship between smoking or obesity, we have shown that age is independent of phenotype (Supplementary Figures 4 and 7*G*).

Somatic mtDNA mutations are not the sole means to experimentally identify clonal relationships. We and others have previously used somatic genomic mutations to demonstrate clonality in BE and intestinal metaplasia of the stomach.^31^, ^32^, ^33^ However, the level of interpatient heterogeneity of specific genomic mutations and the broad range of genes mutated^34^ made detecting such mutations on a gland-by-gland approach for our study difficult. MtDNA mutations are common (Supplementary Figure 7), particularly considering the size of their genome; however, they do not provide any information on the likelihood of progression to cancer because of their neutral impact on cell behavior.^28^ This excludes any inference that the mtDNA mutation is playing a role in the observed phenotypic evolution in our cases. Determination of clonality will be dependent on the clonal markers used and may result in discordance between mitochondrial and genomic analyses of such. Regardless, this does not compromise the phenotypic clonal relationships presented here, but it may result in an underestimation of clone size. Next-generation whole-genome sequencing could address any potential discordance; however, mtDNA Sanger sequencing was deemed a more practical method of clonal analysis when coupled with small amounts of LCM material.

Our clonal analysis data suggest that the cardiac-type gland is the fulcrum on which all other gland types observed in BE are based. Cardiac-type glands are characterized as simple glands, containing only foveolar cells at their surface and mucous-secreting cells at their base. It has been suggested that the presence of non–goblet cell–containing columnar-lined esophagus is associated with shorter lengths of BE and a lower cancer risk.^35^ However, we have previously shown that columnar-lined esophagus can evolve to cancer and can contain oncogenic driver mutations in genes such as *TP53*.^7^ We therefore propose that evolution of cardiac-type glands is the initial basis of progression within BE.

The presence of multiple gland phenotypes across the BE segment may yield important information on the risk of progression to dysplasia or cancer. In terms of physical size, BE changes very little, if at all, over time.^36^ This belies the rate of clonal evolution within the BE segment itself, in particular those patients who are at risk of developing cancer.^16^ Somatic genetic alterations and their diversity in particular are increased in BE before the onset of cancer.^15^, ^16^, ^17^ Controversy surrounds the evolution of genetic diversity, with some studies showing that this occurs only 2–4 years before the onset of cancer,^17^ whereas others indicate that BE from patients who progress always has increased genetic diversity.^15^ This applies to multiple cancer types.^37^ Here, phenotypic diversity is shown to be increased in the nondysplastic areas of BE surrounding dysplasia. We considered that analyzing biopsy specimens may underestimate SIs. Bootstrapping our sampling data, however, showed that a stable Shannon diversity index was achieved with the number of samples analyzed. Although our data support the hypothesis that increased diversity of metaplastic phenotype adjacent to dysplasia reflects overall lesion diversity (as supported by our extensive data sampling of nondysplastic biopsy specimens in Figure 1), we were not able to collect sufficient specimen material to represent the entire length of the Barrett lesion in those patients who had undergone EMR. Although our data cannot completely exclude the possibility that the presence of a dysplastic lesion drove the increased gland diversity in its neighboring mucosa, we consider this less likely because our data show that biopsy specimens taken before the onset of dysplasia also showed a diverse epithelial landscape, suggesting that phenotypic diversity arose before the onset of dysplasia. Indeed, data from the patients with neo-BE suggest that no phenotypic diversity is present in recently developed BE and that this is therefore acquired rather than inherent. Furthermore, in cases that progress to dysplasia, maximal genetic diversity has been shown to occur toward the gastroesophageal junction.^38^^,^^39^ Our samples taken in this same region also show phenotypic diversity regardless of segment length. Phenotypic diversity in BE and other conditions^40^ in the progression to cancer is understudied, and when we consider that tissue diagnoses rely on phenotypic analysis, it is surprising that diversity is not investigated more often.

Overall, this suggests that BE gland phenotypes are diverse; the presence of individual phenotypes are not related to the size of the lesion and can be observed simultaneously in the same location within the esophagus. Our data show definitively that BE phenotypes represent an evolutionary process and suggest that diversity of gland phenotype may play a role in progression.

## Figures and Tables

**Supplementary Methods Figure 1:**
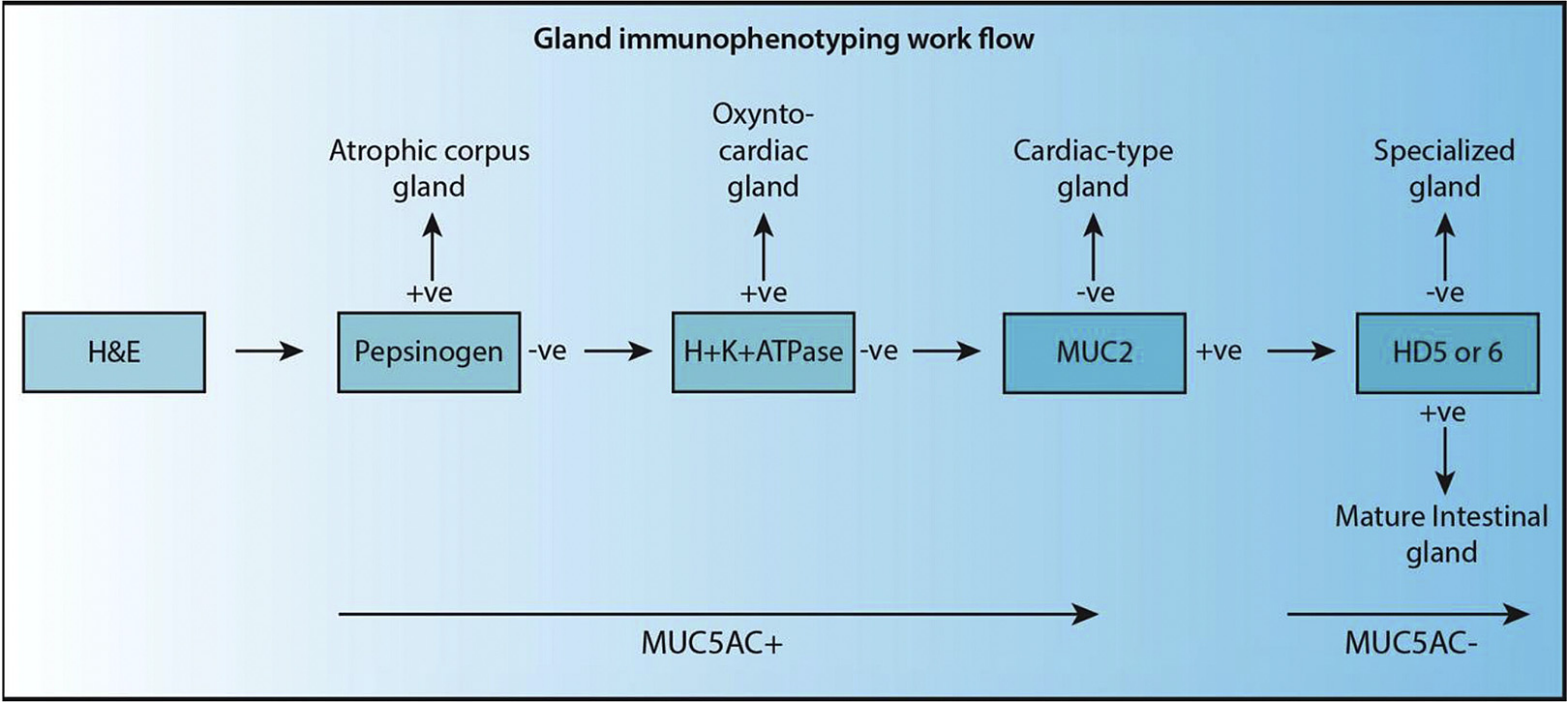
H&E and IHC workflow using specific antibodies to identify gland phenotypes. H&E is the primary histologic guide. Only intestinal glands do not express MUC5AC; however, glands transitioning from specialized to a mature intestinal phenotype may have weak MUC5AC (MUC5AC^lo^) staining and are considered specialized. -ve, negative; +ve, positive.

**Supplementary Table 1:** Clinical Details of Cohort 1a

Patient code	Biopsy code	Prague	Sex	Age at scope, *y*	Distance from GEJ, *cm*	Hiatus hernia,^a^*cm*	Smoking status	Body mass index, *kg/m*^*2*^
107	RH19	C0M3	F	73	0.5	3	N/A	N/A
108	JE41	C7M8	M	49	1	5	N/A	N/A
	JE119	C6M8		50	1	6		N/A
	RH41	C7M9		52	1	6		N/A
	RH82	C8M8		53	1	7		N/A
110	JE48	C0M2.5	M	69	1	3	N/A	28.8
111	JE105	C0M1.5	M	49	1	3	Yes	N/A
	RH05	C2M2		49	2	4		35.6
118	RH26	C9M9	M	55	1	9	N/A	29.5
120	JE120	C8M9	F	83	1	5	N/A	23.4
122	RH38	C0M4	M	83	1	4	N/A	23.4
126	JE125	C2M5	M	50	1	2	N/A	N/A
127	RH60	C14M14	M	64	1	4	Yes	37.5
133	JE29 B6	C5M7	M	89	1	Yes	No	27.8
136	JE32P	C0M0.5-1	M	73	1	Yes	Ex	36.2
137	JE33 B5	C2M6	M	61	1	Yes	Yes	24.2
140	JE36 B5	C12M12	M	59	1	8 to 9	No	30.7
	RH45	C14M14		62	1	9		N/A
	RH72	C12M12		62	1	10		N/A
141	JE37 B5	C0M2	M	56	1	N/A	Yes	N/A
	RH28	C0M2		58	1	2		23.7
143	JE39	C4M5	M	79	1	N/A	Ex	25.6
144	RH31	C0M4	F	74	1	5	Yes	31.0
145	JE42	C2M4	F	62	1	4	No	22.0
	JE111	C1M4		63	1	N/A		N/A
147	JE44	C0M2	M	51	1	N/A	No	31.4
148	JE45	C1M2.5	M	68	1	4	N/A	N/A
	RH20	C0M4		70	1	10		N/A
152	JE50	C2M3	M	27	1	4	N/A	N/A
154	JE52	C0M2	F	52	1	Yes	N/A	N/A
155	JE53	C1M3	M	66	1	6	N/A	N/A
	JE71	C0M2		67	1	4		31.8
157	JE55	C1M5	M	64	1	5	No	35.0
158	RH27	C0M3	M	67	1	N/A	Yes	29.8
160	JE58	C1M5	M	56	1	N/A	Yes	35.0
	RH30	C0M5		58	1	2		N/A
161	JE59	C0M2	F	77	1	Yes	Yes	28.3
165	JE64	C5M6	M	61	1	N/A	Yes	N/A
167	RH32	C0M2	M	51	1	3	No	30.3
168	JE67	C3M7	M	48	1	3	N/A	N/A
172	JE72	C7M8	M	68	1	4	Yes	30.5
174	JE74	C0M4	M	59	1	2	Yes	22.2
178	JE78	C0M5	M	72	1	5	Ex	36.0
179	JE80	C1M2	M	66	1	3	No	19.6
	RH71	C1M3		68	1	3		N/A
180	JE81	C1M6	M	66	1	5	N/A	35.0
	RH91	C0M5		69	1	3		N/A
191	RH12	C8M9	M	60	2	5	N/A	N/A
197	RH14	C7M7	F	68	1	8	N/A	N/A
204	JE113b	C7M8	M	80	1	5	Yes	27.3
205	JE115	C0M2	F	64	1	2	Ex	26.1
207	JE117	C7M8	F	73	1	4 to 5	Ex	N/A
208	JE118	C1M2	M	66	1	N/A	N/A	23.3
213	RH03	C3M4	F	56	1	3	No	26.7
214	RH06	C0M5	M	53	1	N/A	No	31.6
224	RH29	C2M5	M	62	1	4	No	33.1
225	RH33	C0M3	M	62	1	5	No	24.1
227	RH39	C1M4	M	45	1	3	N/A	29.1
230	RH59	C2M4	M	45	2	7	No	29.6
240	RH73	C0M4	M	58	1	2	Ex	26.5
243	RH76	C3M3	F	69	2	7	No	31.2
244	RH77	C7M7	M	68	1	6	Yes	27.2
246	RH85	C4M6	M	72	2	5	Ex	N/A
247	RH88	C5M7	M	56	1	2	Ex	22.3

ex, ex-smoker; F, female; GEJ, gastroesophageal junction; M, male; N/A, not available; no, never smoked; yes, smoker.

a Yes indicates hiatus hernia present but length unknown.

**Supplementary Table 2:** Clinical Information of Cohort 1b

Patient code	Biopsy code	Prague	Sex	Age at scope, *y*	Study	Smoking status	Hiatus hernia,^a^*cm*	Body mass index, *kg/m*^*2*^
133	JE29 B4	C5M7	M	89	Sanger	No	Yes	27.8
180	JE81 B2	C1M6	M	66	Sanger	N/A	5	35.0
146	JE43 B2	C5M6	M	67	Sanger	N/A	N/A	N/A
107	JE01 squamous	C2M3	F	70	NGS	No	No	N/A
	JE01 BE							
	JE01 cardia							
110	JE04 squamous	C2M2	M	69	NGS	N/A	Yes	28.83
	JE04 BE							
	JE04 cardia							
119	JE14 squamous	C3M7	M	62	NGS	N/A	2	29.23
	JE14 BE							
	JE14 cardia							
122	JE17 squamous	C1M4	M	79	NGS	N/A	4	23.4
	JE17 BE							
	JE17 cardia							
123	JE18 squamous	C2M4	M	32	NGS	N/A	4–5	29.39
	JE18 BE							
	JE18 cardia							
126	JE21 squamous	C1M5	M	48	NGS	Ex	2	N/A
	JE21 BE							
	JE21 cardia							
135	JE31 squamous	C3M4	F	81	NGS	N/A	N/A	N/A
	JE31 BE							
	JE31 cardia							
143	JE39 squamous	C4M5	F	79	NGS	Ex	N/A	25.7
	JE39 BE							
	JE39 cardia							
108	JE41 squamous	C7M9	M	49	NGS	No	5	29.09
	JE41 BE							
	JE41 cardia							
149	JE46 squamous	C0M5	M	73	NGS	N/A	3	N/A
	JE46 BE							
	JE46 cardia							

ex, ex-smoker; F, female; M, male; N/A, not available; no, never smoked; yes, smoker.

a Yes indicates hiatus hernia present but length unknown.

**Supplementary Table 3:** Characteristics of the Primary Antibodies Used for IHC

Antibody	Source	Host/isotype	Dilution FFPE	Dilution FF	Secondary	Tertiary
Pepsinogen	Abcam	Mouse/IgG1	1:700	N/A	Goat anti-mouse	Streptavidin AP/blue
H^+^K^+^ATPase	DAKO	Mouse/IgG1	1:1000	1:4000	Goat anti-mouse	Streptavidin HRP/DAB
MUC5AC	Abcam	Mouse/IgG1	1:500	1:50/1:100	Goat anti-mouse	Streptavidin HRP/DAB
MUC2	Thermo Fisher Scientific	Mouse/IgG1	1:500	1:200	Goat anti-mouse	Streptavidin AP/blue
Defensin6α	Sigma	Rabbit/polyclonal	1:5000	N/A	Swine anti-rabbit	Streptavidin AP/blue
Defensin5α	Abcam	Mouse/IgG1	N/A	1:100	Rabbit anti-mouse	Streptavidin HRP/DAB
MUC6	Abcam	Mouse/IgG1	1:500	N/A	Goat anti-mouse	Streptavidin HRP/DAB

NOTE. Antibody sources, dilutions, and processing used in this article.

FF, fresh frozen; N/A = not applicable (antibody not used for this purpose).

**Supplementary Table 4:** Primer Sequences for Whole mtDNA Amplification

Primer	Sequence 5′ → 3′	Position of amplicon in mitochondrial genome
MTL1-F	AAAGCACATACCAAGGCCAC	9397–1892
MTL1-R	TTGGCTCTCCTTGCAAAGTT	
FRAG1-F	TATCCGCCATCCCATACATT	15195–9796
FRAG1-R	AATGTTGAGCCGTAGATGCC	

## Supplementary Methods

### Mitochondrial Next-Generation Sequencing

A total of 30 samples collected from 3 different anatomic sites, across 10 patients (Supplementary Figure 7) were included in the study. For each biopsy, we microdissected 2 × 10^6^ μm^2^ of tissue in 0.5 mL UV-irradiated tubes adhesive caps (Zeiss Technologies). DNA was then extracted using the QIAamp DNA extraction kit (Qiagen) according to the manufacturer’s instructions with the addition of 1 μL of carrier RNA to increase the yield of DNA. Each sample was eluted in 60 μL of nuclease-free water. Extracted DNA was quantified using the Qubit double-stranded DNA High Sensitivity Assay kit (Life Technologies).

The whole mtDNA genome was analyzed using a PCR strategy by generating 2 overlapping amplicons: MTL-1, 9.1 kilobases, and FRAG1, 11.2 kilobases (primers listed in Supplementary Table 4). To minimize PCR errors, the entire mitochondrial genome was sequenced in duplicate independently from each other. The PCR was performed using the TaKaRa LA TaqDNA polymerase kit (Clontech), and the reaction was performed in a volume of 25 μL containing 1× PCR buffer, 0.4 μmol/L primers, 0.4 mmol/L dNTPs, 1.25 units of TaKaRa LA Taq, and 8 μL of template DNA, and the following PCR protocol was used: 94°C for 5 minutes, 33 cycles of 98°C for 15 seconds, 68°C for 10 seconds (slow ramp from 68°C to 60°C at 0.2°C/second) followed by primer annealing at 60°C for 15 seconds and then extension at 68°C for 11 minutes, and a final elongation at 72°C for 10 minutes.

A qualitative and quantitative analysis of the PCR products was performed using a 2200 Tapestation instrument (Life Technologies). All samples were normalized to 0.2 ng/μL and 5 μL (1 ng) of DNA was used for generating libraries with the NexteraXT DNA Sample Preparation Kit (Illumina) according to the protocol. Libraries were then sequenced via the Illumina MiSeq platform v2, 300 cycles (150 nucleotide paired end). Sequencing data have been deposited at the EGA, which is hosted by the EBI and the CRG, under accession number EGAS00001005729. Further information about EGA can be found at https://ega-archive.org.

### Mutation Calling for Next-Generation Sequencing

Data were processed using FastQC, and the average read depth was obtained for each independent BE, cardia, or squamous sample. Samples with low mean read depths (approximately <3500) were resequenced. All variant calls not shared between technical replicates were discarded, and single-nucleotide variants (SNVs) with a variant frequency of <1% were also discarded. Reads were aligned to the revised Cambridge Reference Genome (GenBank accession number NC_012920.1) using the Burrow's-Wheeler Aligner - Maximal Exact Match algorithm^1^ with a default gap open penalty and gap extension penalty of 6 and 1, respectively. For compatibility with Picard’s MarkDuplicates tool,^2^ shorter split hits were marked as secondary during read alignment. Aligned reads were then sorted by coordinate using Picard’s SortSam tool, and duplicates were marked and removed with Picard’s MarkDuplicates tool. SNVs were identified using the deepSNV R package^3^. Bases with a Phred quality score of less than 20 (corresponding to an error probability of *P* = 1% in the original base call) were not considered in SNV calling. A Benjamini-Hochberg correction was applied for multiple testing, and SNVs with a *P* value of less than .05 were considered statistically significant. Rare cases were identified in which sequences (up to approximately 10 base pairs in length) of contiguous mtDNA deletions were detected by deepSNV. These were removed manually from the somatic SNV data on the basis that they are likely to be the result of 1 single deletion, rather than multiple independent SNV events.

Distribution of mitochondrial SNVs was presented as individual Circos plots for each tissue site. Statistical comparison between anatomic sites was performed using a 2-sided paired Student *t* test.

### Spearman Correlation of Richness

The Spearman correlation between richness of phenotype data and the number of biopsy specimens was measured using the Hmisc package (version 4,3,0) in R, implementing a bootstrapping strategy. Each patient (index i) has n_i biopsy specimens. A richness score was calculated by the presence of unique phenotypes over the set of biopsy specimens, that is, R(n_i). The richness R was calculated (R(n_j)) for patient i, and a random subset of biopsy specimens, ranging from j = 1, 2, …, n_i. Each random subset obtained is a single bootstrap replicate and was repeated 1000 times. R(n_j) was calculated for each patient on their subsetted biopsy specimens. The Spearman rank correlation coefficient of R(n_i) was calculated between the number of biopsy specimens sampled and the richness score R(n_j) for each of the 1000 replicates for each patient. The final coefficient values across 22 patients (excluding patients with unchanging richness across all biopsy specimens) were ranked from low to high before visualization using the ggplot2 package (version 3.2.1) in R.

To ensure that sufficient sampling was achieved for diversity per biopsy between nondysplastic BE and BE adjacent to dysplasia, biopsy specimens from each disease state were independently subsampled 1000 times with a random number of glands in each subset (each subset being a bootstrap). Each individual patient’s data were plotted separately.
